# Functional Insights of Salinity Stress-Related Pathways in Metagenome-Resolved *Methanothrix* Genomes

**DOI:** 10.1128/aem.02449-21

**Published:** 2022-04-28

**Authors:** Maria Cristina Gagliano, Pranav Sampara, Caroline M. Plugge, Hardy Temmink, Dainis Sudmalis, Ryan M. Ziels

**Affiliations:** a Wetsus – European Centre of Excellence for Sustainable Water Technology, Leeuwarden, the Netherlands; b Laboratory of Microbiology, Wageningen University and Research, Wageningen, the Netherlands; c Civil Engineering, University of British Columbia, Vancouver, British Columbia, Canada; d Department of Environmental Technology, Wageningen University and Research, Wageningen, the Netherlands; Kyoto University

**Keywords:** anaerobic digestion, EPS, granular sludge, high salinity, *Methanothrix*, N-glycosilation, isoprenoids, methanogens, osmolytes

## Abstract

Recently, methanogenic archaea belonging to the genus *Methanothrix* were reported to have a fundamental role in maintaining stable ecosystem functioning in anaerobic bioreactors under different configurations/conditions. In this study, we reconstructed three *Methanothrix* metagenome-assembled genomes (MAGs) from granular sludge collected from saline upflow anaerobic sludge blanket (UASB) reactors, where *Methanothrix harundinacea* was previously implicated with the formation of compact and stable granules under elevated salinity levels (up to 20 g/L Na^+^). Genome annotation and pathway analysis of the *Methanothrix* MAGs revealed a genetic repertoire supporting their growth under high salinity. Specifically, the most dominant *Methanothrix* (MAG_279), classified as a subspecies of *Methanothrix_A harundinacea_D*, had the potential to augment its salinity resistance through the production of different glycoconjugates via the N-glycosylation process, and via the production of compatible solutes as N^ε^-acetyl-β-lysine and ectoine. The stabilization and reinforcement of the cell membrane via the production of isoprenoids was identified as an additional stress-related pathway in this microorganism. The improved understanding of the salinity stress-related mechanisms of *M. harundinacea* highlights its ecological niche in extreme conditions, opening new perspectives for high-efficiency methanisation of organic waste at high salinities, as well as the possible persistence of this methanogen in highly-saline natural anaerobic environments.

**IMPORTANCE** Using genome-centric metagenomics, we discovered a new *Methanothrix harundinacea* subspecies that appears to be a halotolerant acetoclastic methanogen with the flexibility for adaptation in the anaerobic digestion process both at low (5 g/L Na^+^) and high salinity conditions (20 g/L Na^+^). Annotation of the recovered *M. harundinacea* genome revealed salinity stress-related functions, including the modification of EPS glycoconjugates and the production of compatible solutes. This is the first study reporting these genomic features within a *Methanothrix* sp., a milestone further supporting previous studies that identified *M. harundinacea* as a key-driver in anaerobic granulation under high salinity stress.

## INTRODUCTION

*Methanothrix* (previously named *Methanosaeta*) is a methanogenic archaeal genus of which the associated members are specialists that utilize only acetate, the precursor for more than half of the methane produced on earth (1). In previous studies, *Methanothrix* species were considered to be sensitive to several inhibiting conditions, such as high concentrations of organic acids, ammonia, hydrogen, among others. (2–4). However, more recently, multiple studies have reported on the central role of *Methanothrix* spp. in maintaining stable ecosystem functioning within perturbed anaerobic bioreactors. For instance, *Methanothrix* spp. were resistant to drastic acetate increases (5, 6), high organic acid and ammonia concentrations (7), and elevated concentrations of the long-chain fatty acid palmitate (up to 4 mmol/L) (2). *Methanothrix* was also found to be the main acetate-degrading genus within stable anaerobic reactors with efficient methane production (6, 8).

With their low excess sludge production, granular sludge systems like upflow anaerobic sludge blanket (UASB) reactors are now a widely adopted option to treat industrial wastewaters (9). *Methanothrix* spp. can constitute the initial nuclei for the formation of such granules (10–13), likely due to their ability to form filaments (13–15) as well as to the specific glycoproteic, hydrophobic sheet surrounding these filaments which stimulates bio-aggregation (16–18). In recent years, there has been a growing interest in the applicability of high-rate anaerobic reactors for treatment of saline wastewaters (19–21), amounts of which are expected to increase globally (22, 23). UASB is a promising biotechnology platform to treat saline industrial wastewater streams, for example before a desalination step for water reclamation or harvesting (24, 25). However, high Na^+^ concentrations can cause cell lysis, inhibit methanogens and disrupt the structure of UASB granules (26, 27). Despite this, *Methanothrix harundinacea* was recently detected in compact and large-sized granules in UASB reactors at elevated salinity levels (from 5 to 20 g/L Na^+^) (19, 28, 29). Pure cultures of *M. harundinacea* have, however, not been described to be tolerant to Na^+^ (30).

*In-silico* analysis of the complete genome of *M. harundinacea* strain 6Ac (31) revealed several genetic loci that potentially encode for salinity-stress related pathways, such as the synthesis of compatible solutes and the production of different surface glycoconjugates constituting the extracellular polymeric substance (EPS) (32). The accumulation of compatible solutes is a well-known osmoprotection strategy by some methanogenic archaea, which mostly synthesize derivatives of β-amino acids, such as β-glutamine and N^ε^-acetyl-β-lysine (33, 34). The secretion of EPS is also recognized as a fundamental microbial adaptation to salinity, providing osmotic tolerance and limiting dehydration (35). The composition of excreted EPS can change in response to salinity fluctuations (36–38), as has been observed in the halophilic archaeon Haloferax volcanii when salinity decreases (39). A similar adaptation strategy observed within halophilic archaea is the production and inclusion of isoprenoid derivatives and carotenoids into lipid membranes, which helps in maintaining their fluidity in response to changes in the osmotic conditions (40). Yet, changing the outer layer structure via the synthesis of different EPS glycoconjugates or isoprenoids are stress responses that have never been reported for methanogenic archaea. Therefore, mechanisms of adaptation to saline wastewater streams in UASB reactors remain unclear, particularly for keystone *Methanothrix* spp. (28, 32).

In our previous work, we demonstrated microbial granulation from dispersed biomass in stable UASB reactors at low salinity (LS) and high salinity (HS) (working at 5 and 20 g/L Na^+^, respectively), with a clear dominance of *M. harundinacea* clusters in the formed granules (28, 29). Using fluorescence *in-situ* hybridization (FISH), lectin staining, and clonal sequencing analysis, we also observed different subtypes of *M. harundinacea* with distinct aggregation behavior and an EPS-glyconjugate pattern shift in response to changes in salinity (32). Additionally, after exposing granules grown at 20 g Na^+^/L to a salinity shock, we identified N^ε^-acetyl-β-lysine as one of the excreted compounds, indicating the presence of anabolic pathways within the bioreactor for its formation (41). Therefore, we hypothesized that the *Methanothrix* genomes within those saline UASB reactors would contain the genetic repertoire to enable the adaptation toward high salinity. In this study, we investigated this hypothesis by analyzing samples taken in a time-series from the same saline UASB reactors using genome-resolved metagenomics. The reconstructed metagenome-assembled genomes (MAGs) were annotated for different salinity-stress related functions, and were compared in a pangenomic analysis with other representative *Methanothrix* related genomes, salinity adapted methanogens, and salinity adapted archaea. The different genomic features identified in this work suggest that a newly discovered *M. harundinacea* subspecies can be considered a halotolerant methanogen, adding support to our previous findings identifying *Methanothrix* as a key-driver in anaerobic granulation under salinity stress.

## RESULTS AND DISCUSSION

### Classification of methanogenic metagenome-assembled genomes.

**FIG 1:**
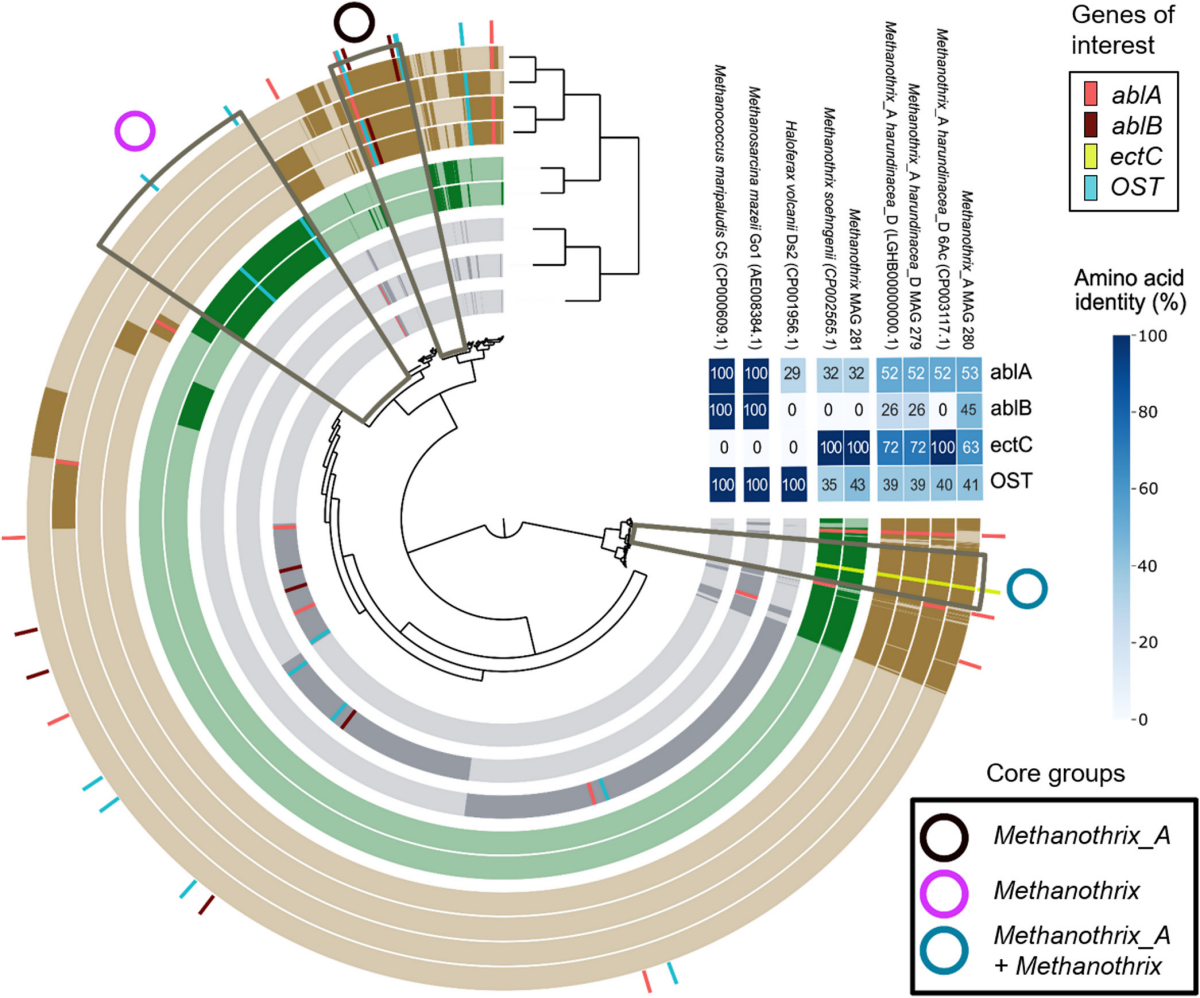
Pangenome analysis of *Methanothrix* related genomes, salinity adapted methanogens and archaea from NCBI database (accession numbers in brackets), along with *Methanothrix*_A *harundinacea*_D MAG_279, *Methanothrix*_A MAG_280, and *Methanothrix sohegenii* MAG_281. The dendrogram on the top represents a hierarchical clustering of the genomes based on presence/absence of genes. The clustering of the genes is based on amino acid sequence similarity, as performed on Anvi’o (v7) using BLASTP. Per each genome analyzed, the core gene groups are identified with darker spots along the pangenome. Within these core groups, key genes related to salinity stress functions (as osmolytes production and EPS excretion) are highlighted (color legend on the top-right). The associated heatmap on the right shows amino acid sequence identity for those genes estimated using BLASTP. The genes are: ablA (l-lysine 2,3-aminomutase), ablB (β-lysine N(6)-acetyltransferase), ectC (L-ectoine synthase) and OST (oligosaccaryl transferases).

Details on the operation and performance of the LS and HS UASB reactors, working at 5 and 20 g/L Na^+^, respectively, are presented by Sudmalis, Gagliano et al. (29), while a preliminary assessment of the microbial community based on 16S rRNA amplicon sequencing and FISH was outlined by Gagliano et al. (28). A summary of the operational and performance parameters of the two reactors is provided in Table S1. In this study, we further analyzed the archaeal community of both UASB reactors by recovering a dereplicated set of MAGs, with a focus on obtaining a higher-resolution understanding of *Methanothrix* population dynamics. Granule samples were collected from both reactors at 10 time points, starting from day 79 (after the first granules had formed in the HS reactor) until the end of the operational period (day 217; Fig. S1). The genome-resolved-metagenomics effort recovered one *Methanothrix* and two *Methanothrix*_A MAGs, based on classification with the Genome Taxonomy Database (GTDB) (42). The two *Methanothrix*_A genomes, MAG_279 and MAG_280 had a completion of 89% and 93%, respectively, and each had a redundancy of 0.7%. The single *Methanothrix* genome, MAG_281, had a completion of 94% and redundancy of 2%. *Methanothrix*_A MAG_279 shared a 99.7% average nucleotide identity (ANI) with *Methanothrix harundinacea* 56_747 (accession number LGHB00000000.1) (Fig. S2), likely belonging to the same species (43). *Methanothrix*_A MAG_280 had a maximum ANI of 88.9% with *M. harundinacea* isolate UBA475 (accession number DAXT00000000.1) across 60 *Methanothrix*_A and *Methanothrix* genomes available from the NCBI database (Fig. S2), suggesting that it may be a novel species based on an ANI cutoff of 95% (43). Finally, *Methanothrix* MAG_281 shared an ANI of 99.2% with *M. soehngenii* AS27yjCOA_157 (accession number JAAYUN000000000) (Fig. S2). The three MAGs encode the complete pathway for acetoclastic methanogenesis (Fig. S3), in agreement with the known ecological niche of *Methanothrix* (1). The recovered *Methanothrix* MAGs were compared in a pangenome analysis based on amino acid sequence similarity of genes, along with two representative *Methanothrix harundinacea*, two salinity adapted methanogens (Methanosarcina mazei Go1 and Methanococcus maripaludis C5) (44, 45) and the halophilic archaeon Haloferax volcanii Ds2 (46). The pangenome analysis identified 12,306 gene clusters (Fig. 1), from which three “core groups” were distinguished based on genes being systematically detected and clustered together within *Methanothrix* taxonomic groups: (i) the *Methanothrix*_A core included 610 gene clusters; (ii) the *Methanothrix* core contained 1,030 gene clusters; and (iii) the shared core by *Methanothrix*_A and *Methanothrix* constituted 418 gene clusters (Fig. 1). The *Methanothrix_A* core gene group contained a higher number of gene families connected to salinity stress in comparison to other *Methanothrix* lineages (Fig. 1). Gene clusters encoding for salinity stress functions were also found in the accessory genomes of *Methanothrix* and *Methanothrix_A*, but they were grouped separately from those of *M. mazei*, *M. maripaludis*, and H. volcanii, indicating a potentially unique response of the *Methanothrix* ssp. identified in this study (Supplementary Data File 1). Salinity stress-related related functions were further investigated within the reconstructed *Methanothrix* MAGs in relation to the community dynamics observed during the reactor operation, as discussed in the following sections.

### Dynamics of Methanothrix spp. in low and high salinity anaerobic reactors.

**FIG 2:**
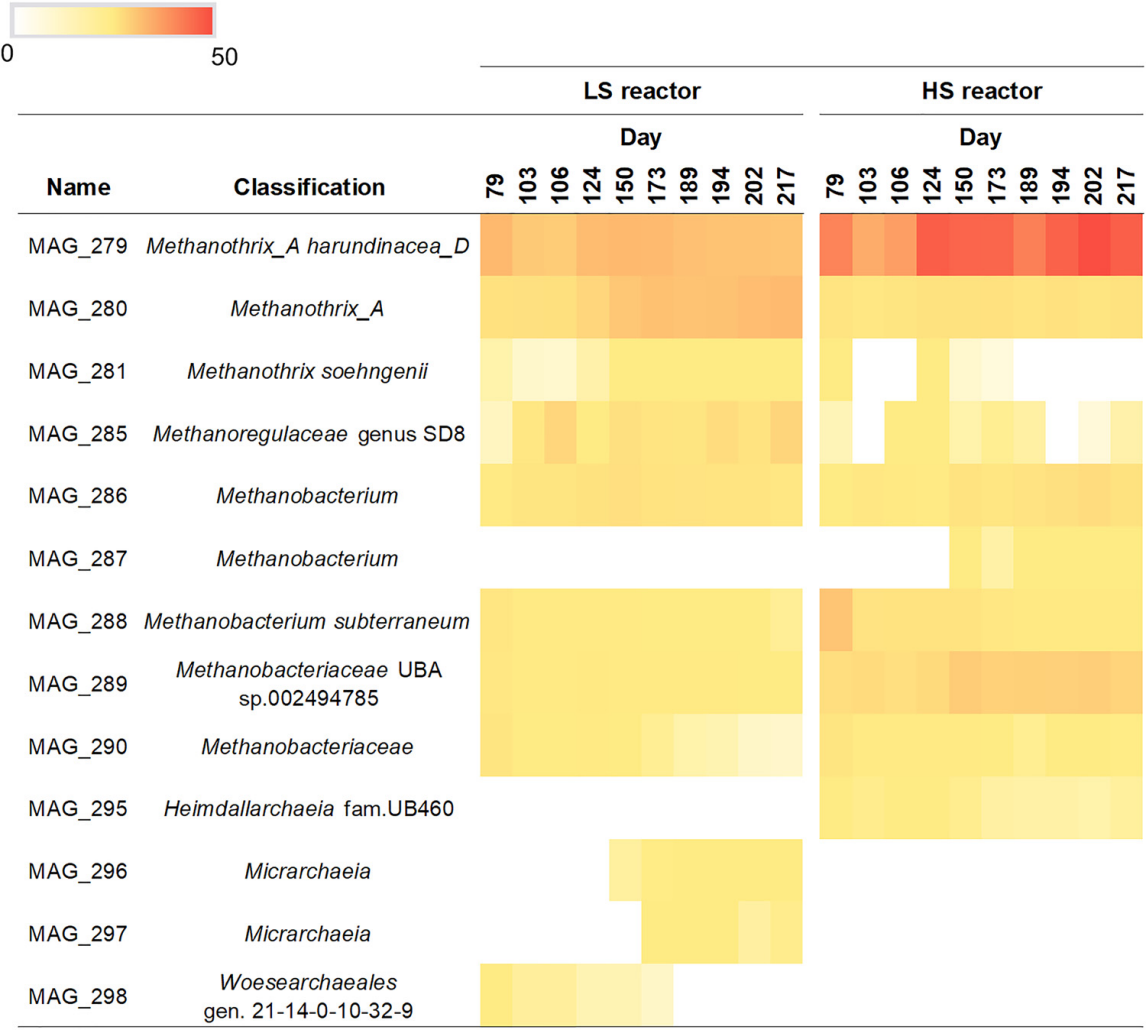
Heatmap showing the relative abundance of de-replicated archaeal MAG in the LS (5 g/L Na^+^) and HS (20 g/L Na^+^) reactors, reported as a percentage (in the range 0% to 50%) out of the total microbial population, from day 79 until the end of the anaerobic process operation (day 217).

**FIG 3:**
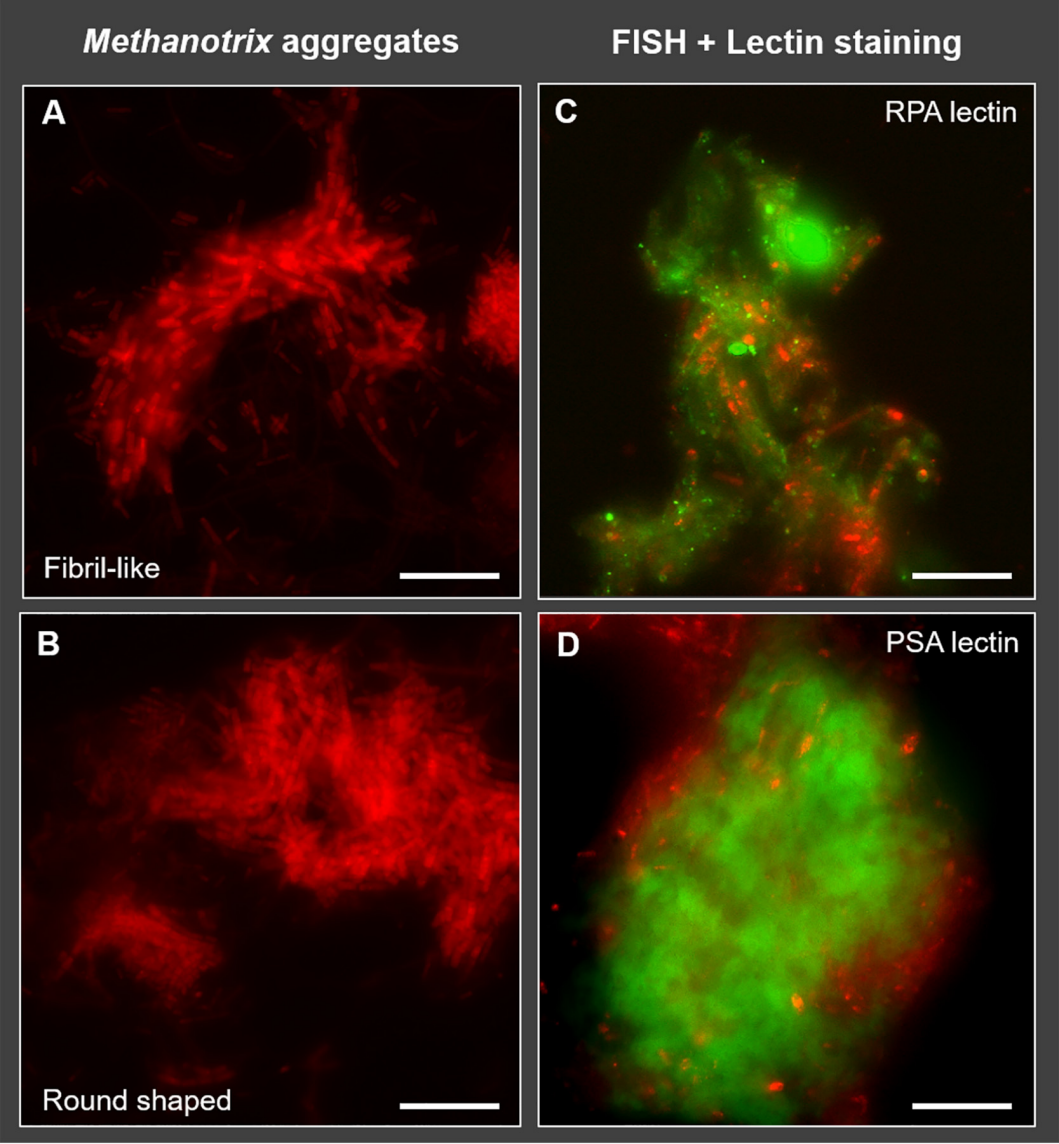
In (A) and (B), epifluorescence images of the two kind of *Methanotrix* aggregated clusters visualized by FISH with probe ARC915 in HS granules. In (C) and (D), epifluorescence images the same samples stained using RPA (specific for N-Acetyl Galactosamine rich glycosides) and PSA (specific for branched Mannose) lectins combined with probe MSMX860 (*Methanosarcinales*), which proved the association of the two *Methanothrix* shapes with different exopolymeric structures. Size bar is 10 μm.

While the two *Methanothrix_A* and one *Methanothrix* MAGs were detected within both the LS and HS reactors, their abundance differed between the two systems (Fig. 2). *M. harundinacea* MAG_279 was the dominant archaeon under both salinity conditions, with an average relative abundance 12% in LS and 33% in HS, out of the total microbial population (Fig. 2). *M. harundinacea* MAG_279 remained the dominant *Methanothrix* sp. within all 10 time points analyzed for the HS reactor, while it had a similar relative abundance as *Methanothrix*_A MAG_280 in the LS reactor from around day 173 until the end of the experimental period (between 11% and 13%) (Fig. 2). The relative abundance of *Methanothrix*_A MAG_280 in the HS reactor was substantially lower and constant between 2% to 3% (Fig. 2). The abundance of *M. soehngenii* MAG_281 was negligible within both reactors (< 1%), consistent with the lack of literature reporting salinity-adapted strains of this species. These results confirm that *M. harundinacea* MAG_279 out-competed *Methanothrix*_A MAG_280 and *M. soehngenii* MAG_281 for acetate under highly saline conditions up to 20 g/L of Na^+^. Furthermore, the ANI analysis revealed that *M. harundinacea* MAG_279 shared the highest ANI with Methanosaeta harundinacea 56_747 (GCA_001508615.1) (Fig. S2), which was identified in sediment samples collected within an oil field subsurface reservoir (47). The start-up inoculum from which *Methanothrix*_A MAG_279 originated was from a full-scale UASB reactor treating industrial wastewater rich in salt (≈8 g/L Na^+^) produced by the Shell plant in Moerdijk, the Netherlands. In both of these systems, seawater is pumped into the system either for oil recovery or in the cooling towers within the petrochemical plant, likely stimulating salt adaptation of the resident microorganisms. With the taxonomic classification performed using the GTDB (42), *M. harundinacea*, as all the other members of the order *Methanosarcinales*, is classified under the phylum *Halobacterota*. This phylum was proposed in an extensive study of genome phylogenies utilizing a concatenated alignment of 122 marker proteins for archaea (48) and includes the class *Halobacteria*, within which most salt-requiring and salt-resistant archaea are grouped. However, due to the profound changes that the archaeal phylogeny and taxonomy has undergone in recent years (49–52), the adaptation of these *Methanothrix* spp. to salinity could have occurred independently from the origin of their phylogenomic classification. Through the application of FISH on both granule types (28, 32) we found that *Methanothrix* cells were aggregated in two sorts of clusters: fibril-like shaped, with short filaments approaching each other (Fig. 3A); and round shaped, rich in rods (Fig. 3B). We hypothesized that the two shapes of clusters could represent different subspecies of *M. harundinacea*, as was also indicated by the wide range of similarities observed among *Methanothrix*-affiliated 16S rRNA genes detected via clonal analysis on DNA samples extracted from same reactors granules (28). Our findings here suggest that the round and fibril-like clusters could have in fact been comprised of two different *Methanothrix_A* species, MAG_279 and MAG_280. *Methanothrix* spp. indeed tend to aggregate as compact clusters under stress conditions, as previously observed within granules grown at thermophilic temperatures (53, 54). Both aggregation behaviors were particularly visible in HS granules, where the application of FISH combined with lectin staining highlighted different EPS and glycoconjugate patterns/structures (capsular and cloudy EPS) surrounding these *Methanothrix* clusters (Fig. 3C and D). In addition to this physiological evidence, the genome-resolved metagenomics and functional annotation of the MAGs performed here identified several genetic loci that are potentially involved in the production and modification of surface glycoconjugates, as discussed below.

### Protein glycosylation and outer layer modifications functions.

**FIG 4:**
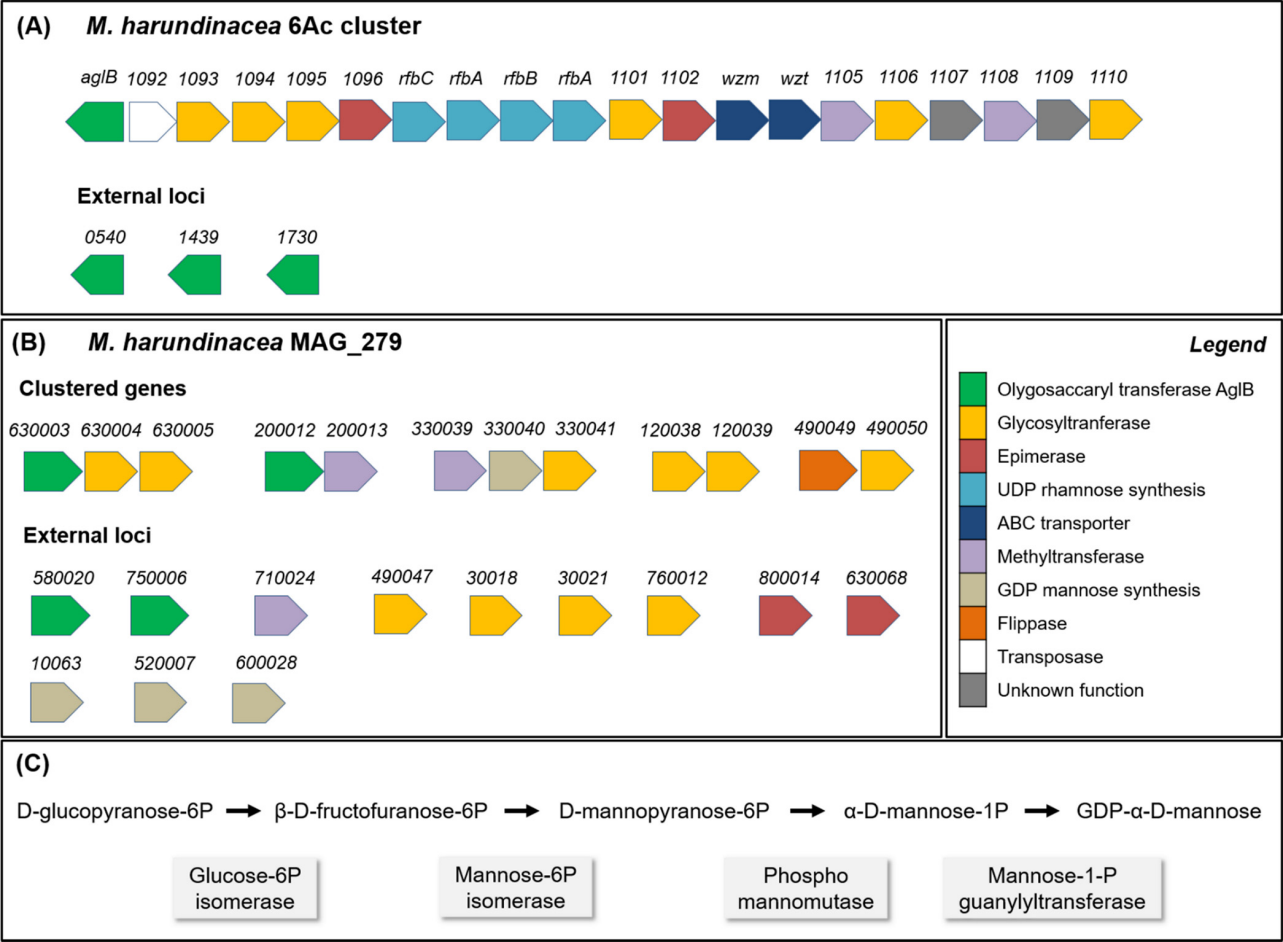
Schematic representation showing the N-glycosylation genes organization in *M. harundinacea* 6Ac (A) and in *M. harundinacea* MAG_279 (B). In the *M. harundinacea* 6Ac genome, a cluster of 20 genes for the N-glycosylation pathway is present (loci 1091 to 1110), resembling the one of H. volcanii. In MAG_279 instead, the putative N-glycosylation genes were grouped into small clusters. In (C), the mannose synthesis pathway as identified in MAG_279.

The production of EPS has been identified in different groups of archaea, predominantly in halophiles and thermophiles (55), but not yet in methanogens. Proteins and carbohydrates in EPS are often combined in the form of glycoconjugates (56). N-glycosylation is one of the most prevalent protein modification processes at the microbial surface layer (S-layer), in which sugars are covalently attached to asparagine residues (57). In O-glycosylation, carbohydrates are instead transferred to serine or threonine residues, but this mechanism has been less studied within the domain *Archaea* (58, 59). Archaea have unique N-glycosylation features in comparison to eukaryotes and bacteria, including the ability to vary the N-glycan composition under different growth conditions (57, 60). For instance, N-glycosylation of S-layer proteins is a salinity response in the archaeon H. volcanii, and at least three N-glycan structures have been identified when this microorganism is growing at different salt concentrations (57). While under high salinity stress (3.5 M NaCl), H. volcanii was observed to decorate the N-glycans with mannose as the sugar, at lower salinity (1.75 M NaCl) a different pathway was observed, involving rhamnose as the sugar on the glycosylated proteins (39). This ability to change glycosylation patterns likely relies on the presence of multiple copies of the gene for the oligosaccharyl-transferase (OST) AglB, which is responsible for delivering glycan to target protein asparagine residues. The presence of multiple AglB-encoding sequences within a single genome could potentially provide different specificities for different glycan moieties (61). In our previous work, we showed that the EPS surrounding the round-shaped methanogenic cells clusters in the UASB granules shifted from a thick outer layer, identified at low salinity, to a cloud-like EPS structure at high salinity (32) (Fig. S4). These EPS shapes were composed of similar sugars, which differed in the arrangement of their glycoconjugate patterns (description in Fig. S4). The cloud-like EPS was found to be rich in branched mannose, of which the likely function was to bind and “inactivate” Na^+^, shielding the *Methanothrix* cells from excess intake (32, 62). Mannose is the major component of the EPS excreted by many halophilic archaea, such as Haloferax mediterranei (63), H. volcanii (64), and Haloarcula japonica (65), or other extremophiles like Thermococcus litoralis (66). In N-glycosilation by H. volcanii, several glycosyl transferases (GT) are responsible for sequentially adding carbohydrate residues onto a dolichol phosphate (DolP) carrier on the inside of the cell to build up a glycan. Then, a methyltransferase (MT) catalyzes the addition of a methyl group, a flippase delivers the DolP-bound glycan across the plasma membrane, and finally an OST (AglB) transfers it to the target asparagine residue (67). An *in-silico* analysis of the *M. harundinacea* 6Ac genome revealed the presence of a cluster coding for an N-glycosylation pathway resembling that of H. volcanii (32, 39) and multiple copies of the OST AglB, associated with a sub-cluster for rhamnose synthesis (Fig. 4A). In *M. harundinacea* MAG_279, we detected multiple genes encoding for GT, as well as three genes for MT, one gene for a flippase, two genes for epimerase, and four genes encoding for the OST AglB putatively involved in the N-glycosylation (Fig. 4B; Table S2).

By analyzing the four OSTs genes found in *M. harundinacea* MAG_279, we detected two functional domains able to catalyze either N- or O-glycosilation of membrane proteins. One is an archaeo STT3 (archaeosortase A system-associated) domain, belonging to the subfamily of AglB (Table S3), which catalyzes the transfer of a defined glycan from DolP- to an asparagine residue. The STT3 domain occurs in the phylum *Euryarchaeota*, and particularly within the orders *Archaeoglobales* and *Halobacteriales* (including H. volcanii*)*, as well as class II methanogens including the orders *Methanomicrobiales* and *Methanosarcinales* (including *M. harundinacea)* (68). The second domain belongs to the PMT_2 superfamily, which encodes for the dolichyl-phosphate-mannose-protein mannosyltransferase (PMT) that catalyzes the transfer of a mannose from Dol-P-mannose on serine or threonine residues (69) (Table S3). In addition, all of the genes for a putative N-glycosilation pathway were detected in *Methanothrix_A* MAG_280 (Table S2). The three OST genes identified in MAG_280 also encoded for enzymes with STT3 and PMT2 domains, but they were distinct from those of MAG_279 (Table S3), which could also explain the observed differences in their physiologies at high salinity. Moreover, the amino acid identity between the OSTs in *Methanothrix*_A MAGs and other salinity-adapted methanogens (*M. maripaludis* C5 and *M. mazei* Go1) was about 40% (Fig. 1), and such distinct amino acid signature could indicate the potential unique specificity of *Methanothrix*_A OSTs in their salinity-stress related glycosylation pathways (Fig. 1). Methanogens do not assimilate carbohydrates, but rather rely on gluconeogenesis for their formation (e.g., pentoses and hexoses) (70). All three of the *Methanotrix*-related MAGs possessed the genetic repertoire to produce glucose from pyruvate via gluconeogenesis through the same Embden-Meyerhof-Parnas (EMP) pathway as identified in Methanococcus maripaludis (Fig. S5) (70, 71). As observed in the archaeon Pyrococcus horikoshii OT3 (72), the GDP-mannose required for the synthesis of Dol-P-mannose from glucose-6-phosphate could be produced by MAG_279 and MAG_280 via the sequential action of a glucose-6-phosphate isomerase, a phophomannomutase and a mannose-1-phosphate guanyltransferase (Fig. 4C, Fig. S6).

Overall, the presence of different OST-encoding genes, together with the different types of EPS structures detected via lectin staining surrounding the methanogenic clusters at low and high salinity (Fig. S4), strongly suggests that the genes identified in this study are connected to a molecular mechanism were salinity influences the specificities for a variety of glycan assemblies in *M. harundinacea* MAG_279. The putative N-glycosylation genes identified in *M. harudinacea* MAG_279 are distantly located, clustered as two or three open reading frames, differently than in the genome of *M. harundinacea* 6Ac in which a total of 20 N-glycosylation genes are clustered together (Fig. 4A), as also observed in H. volcanii (32, 60). The clustering of N-glycosylation genes together with at least one AglB gene has been shown within the genomes of other archaeal species (60, 61). In H. volcanii, it was demonstrated that most of these clustered genes are transcribed coordinately as a single genomic unit (73, 74). However, the lack of clustered genes putatively participating in the N-glycosylation process is also common, such as in thermophilic archaea (57), and this doesn’t limit the functionality of the pathway.

### Assessing the potential of the *Methanothrix*-related MAGs for isoprenoids biosynthesis.

**FIG 5:**
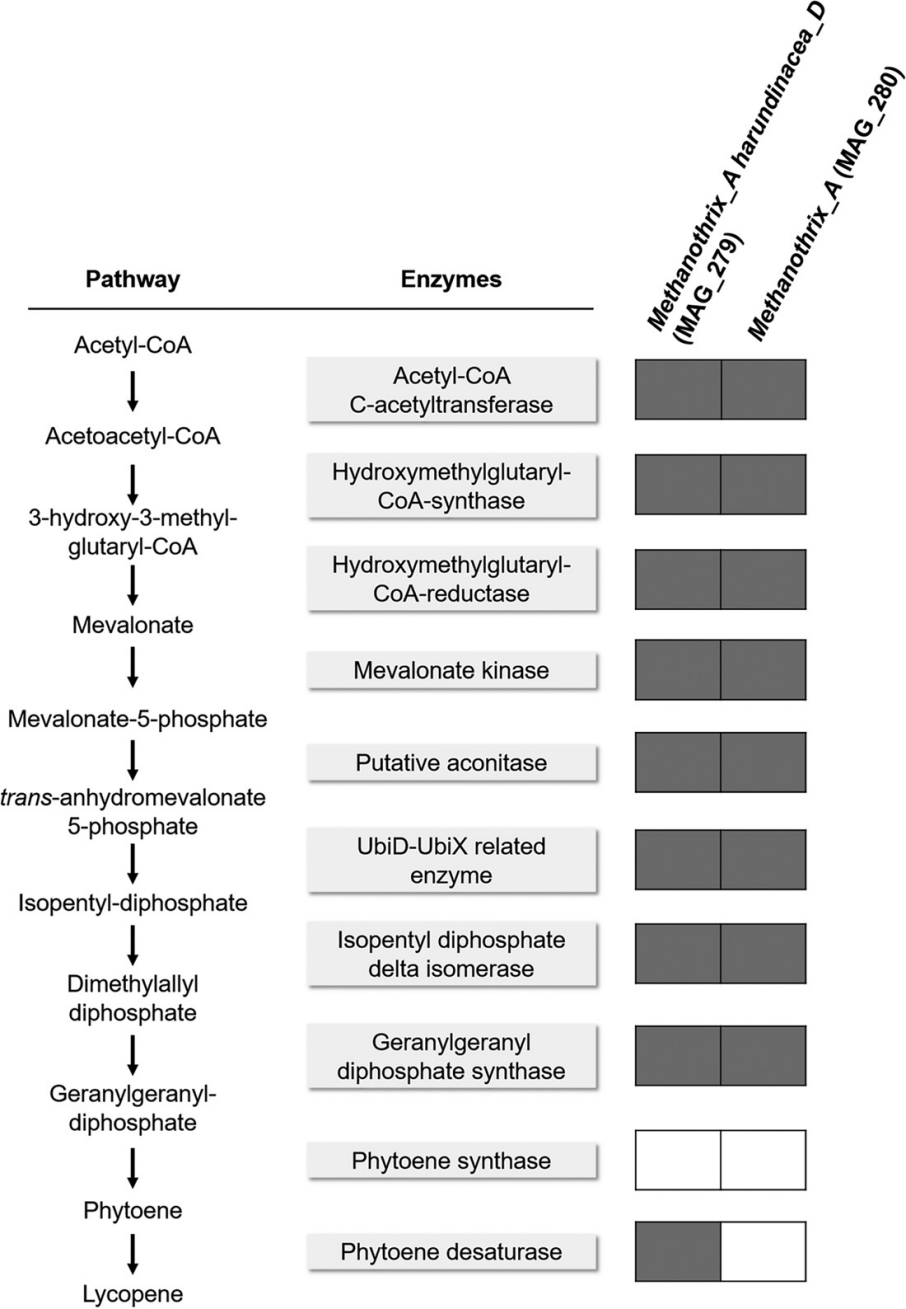
Putative isoprenoids synthesis via an alternative mevalonate pathway and the annotated enzyme functions found in the two salinity adapted *Methanothrix* MAGs reconstructed in this study (MAG_279 and MAG_280). Gray and white squares indicate the presence or absence of the encoding gene.

The synthesis of isoprenoids, such as carotenoids, and their integration in the cell membranes is a metabolic adaptation in haloarchaea, which similarly to the EPS function outlined in the previous paragraph, can act as a barrier in hypersaline conditions (75). For instance, H. volcanii in most cases is pink-red colored, given the production of like bacterioruberin, a C_50_ carotenoid found in the lipid membrane of many other haloarchaea (76). The stress-related accumulation of carotenoids has been well described for microalgae: stress conditions which cause increase in intracellular reactive oxygen species (ROS) can damage to macromolecules such as DNA, lipids, and proteins (77). To scavenge ROS, microalgae synthesize additional carotenoids to form a protective layer that prevents reactive radicals and lipid peroxidation, and thus preserving the cell membrane integrity (78). The precursor of isoprenoids is geranylgeranyl pyrophosphate (GGPP), which in most microorganisms is synthetized via the mevalonate (MVA) pathway through the C-5 intermediate isopentenyl pyrophosphate (IPP) (40, 76). All isoprenoids like carotenoids are synthesized by the consecutive condensation of the C-5 monomer IPP to its isomer, dimethylallyl diphosphate (DMAPP) (79). IPP and DMAPP isoprenoid precursors are fundamental for the synthesis of isoprene-based alkyl chains constituting the archaeal membrane lipids, characterized by ether linkages to the glycerol moieties (80). These lipids and ether linkages are thought to improve membrane stability, providing an advantage in different extreme environments, such as high salinity (81, 82). The genomes of *M. harundinacea* MAG_279 and *Methanothrix_A* MAG_280 encode all of the genes for the biosynthesis of GGPP via the MVA pathway as found in H. volcanii, with the exception of mevalonate-5-phosphate decarboxylase (PMD) (Fig. S7). The gene for PMD is conserved across all haloarchaea, but is not found in most other archaea (83). Indeed, different MVA pathways have been found in other archaea, for instance in *Thermoplasma acidophilus*, Aeropyrum pernix, and *Methanocarcina mazei*, where the function of mevalonate-5-phosphate decarboxylase is substituted by other enzymes/reactions (83–85). In the hyperthermophilic archaeon *A. pernix*, two enzymes can replace the PMD function: (i) a putative aconitase catalyzes the dehydration of mevalonate 5-phosphate to trans-anhydromevalonate 5-phosphate; and (ii) an enzyme belonging to the UbiD-decarboxylase family, together with a UbiX-like partner, converts the intermediate into IPP (83). This *Aeropyrum*-type, modified MVA pathway, seems widely distributed among the domain *Archaea*, mostly among anaerobes, probably because it requires less ATP (83, 85). Through a comparison with putative orthologs of the *A*. *pernix* enzymes contributing to the modified MVA pathway found in Methanothrix thermophila (83), we identified four genes that could correspond to the putative aconitase and the UbiD/UbiX functions in *M. harundinacea* 6Ac, *M. harundinacea* MAG_279, and *Methanothrix_A* MAG_280 (Table S4), and reconstructed the pathway (Fig. 5). In MAG_279 and MAG_280, IPP can be converted to DMAPP via an isopentyl diphosphate delta-isomerase, and DMAPP in turn is converted into GGPP via a geranylgeranyl diphosphate synthase (Fig. 5). GGPP is a C_20_ isoprenoid required for carotenoid synthesis: two molecules of GGPP are condensed to phytoene, which is then converted to lycopene, the precursor of many carotenoids such as bacterioruberin (76). The formation of phytoene is catalyzed by phytoene synthase, which is converted to C_40_ lycopene by phytoene desaturase. Finally, lycopene elongase converts lycopene to C_50_ bacterioruberin (46). We did not identify the set of genes encoding for the bacterioruberin synthesis in MAG_279 and MAG_280, and a gene for phytoene synthase was missing. However, in *Methanothrix_A* MAG_279 we identified a homologue of phytoene desaturase (E-value from HMM search: 8^E-0.5^), which could potentially lead to lycopene formation. A phytoene desaturase-like function was also found be involved in the synthesis of hydroxyarchaeol, a typical core structure of archaeal membrane lipids uniquely detected in a limited number of lineages of the methanogenic archaea, including *M. soehgenii* (86). Hydroxyarchaeol can further stabilize membranes under extreme conditions, but a direct correlation with salinity levels has not yet been demonstrated (87).

### Pathways encoding for the production of osmolytes.

**FIG 6:**
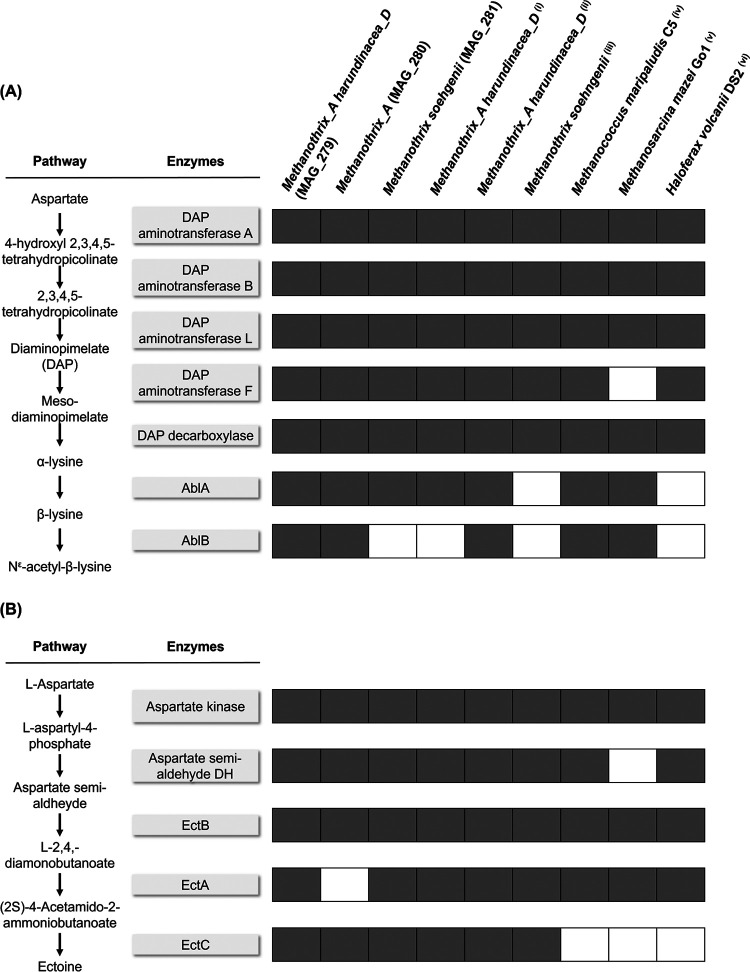
Putative pathways for the synthesis of the compatible solutes N^ε^-acetyl-β-lysine (in A) and ectoine (in B) and the annotated enzyme functions found in the three *Methanothrix* MAGs reconstructed in this study (MAG_279, MAG_280 and MAG_281) in comparison with other 6 genomes of archaea known to have salinity stress-related functions. The NCBI accession numbers of the genomes used for the comparison are: (i) CP003117; (ii) LGHB00000000.1; (iii) CP002565; (iv) CP000609; (v) AE008384; (vi) CP001956. Per each genome analyzed, gray and white squares indicate the presence or absence of the encoding gene.

While the strategies described above prevent the excess accumulation of Na^+^ intracellularly, the production and accumulation of osmolytes can protect intracellular structures from osmotic stress, maintaining the cell volume. The latter is also hypothesized to be a mechanism employed to abate salinity stress by the microbiome of the HS reactor. Indeed, N^ε^-acetyl- β-lysine was previously identified as the main osmolyte excreted by the granules from HS reactor when subjected to an abrupt decrease in salinity from 20 g Na^+^/L to 5 g Na^+^/L (41). Because N^ε^-acetyl- β-lysine is the predominant compatible solute in methanogenic archaea (34), we investigated whether the genomes of *M. harundinacea* MAG_279 and *Methanothrix_A* MAG_280 could encode for its production, together with other osmolytes. Among methanogens, N^ε^-acetyl-β-lysine has been found to be produced by *Methanosarcina* ssp., Methanogenium cariaci, and *Methanohalophilus* FDF1 (88–90), but has never been reported for *Methanothrix* spp. In these methanogens, the synthesis of N^ε^-acetyl-β-lysine is initiated by the production of α-lysine from aspartate semi-aldehyde and pyruvate via the diaminopimelate (DAP) pathway (88, 89). The diaminopimelate aminotransferases (DAP-related enzyme encoded by the genes *dapA*, *dapB*, *dapL*, and *dapF*), sequentially catalyzes aspartate semi-aldehyde to meso-diamonipimelate, that is converted to α-lysine by diaminopimelate decarboxylase. Lysine-2,3-aminomutase (AblA) catalyzes the formation of β-lysine from α-lysine, which is then acetylated to form N^ε^-acetyl-β-lysine by the β-lysine N(6)-acetyltransferase (AblB) (34, 45, 88). The genomes of *M. harundinacea* MAG_279 and *Methanothrix_*A MAG_280 both encode the DAP pathway (Fig. 6, Fig. S8), as is commonly found in other methanogenic archaea for the synthesis of lysine (91). Homologues of the two genes *ablA* and *ablB*, crucial to synthesize N^ε^-acetyl-β-lysine from α-lysine, were detected in both MAG_279 and MAG_280 (Fig. 6, Fig. S8). These genes are typically salt-induced and not active under normal growth conditions (34). Interestingly, the homologue of the *ablB* gene (BLASTP e-value < 10^−4^) observed in *M. harundinacea* MAG_279 and *Methanothrix*_A MAG_280 (Fig. S7) was located in the core gene group of *Methanothrix*_A identified in the pangenome analysis, but it was separately clustered from those found in salinity adapted archaea (Fig. 1; Supplementary Data File 1). Thus, a unique β-lysine N(6)-acetyltransferase function could be encoded by *Methanothrix*_A MAGs compared with other salinity adapted archaea, as also supported by the low amino acid identity for this *ablB* gene in comparison to the other sequences analyzed (26% for MAG_279 and 45% for MAG_280; Fig. 1).

Another pathway related to a compatible solute-based osmoprotection mechanism found in the *Methanothrix*-related MAGs was ectoine synthesis. Ectoine is produced from aspartate in a three-step pathway via a L-2,4-diaminobutyrate transaminase (EctB), a 2,4-diaminobutyrate acetyltransferase (EctA), and an ectoine synthase (EctC) (92). As observed for N^ε^-acetyl-β-lysine, where just two of the enzymes of the pathway (AblA and AblB) are crucial for its production, EctC can be regarded as a diagnostic enzyme to identify ectoine producers (93). In a study analyzing 557 genome sequences of archaea to assess the presence of the ectoine biosynthetic genes, *ectC*-type genes, grouped in an *ectABC* gene cluster, have been detected in two species of the *Methanothrix* genus, *M. harundinacea* 6Ac and *M. soehngenii* GP6 (92). In *M. harundinacea* MAG_279 we identified the three genes *ectA*, *ectB* and *ectC* grouped in a cluster, while in *Methanothrix_A* MAG_280 no homologous of *ectA* were identified (Fig. 6 and Fig. S9). This finding could further explain why *M. harundinacea* MAG_279 had a significant growth advantage over *Methanothrix_A* MAG_280 with the increase of salinity from the LS to the HS reactor.

### Outlooks and conclusive remarks.

The genome-centric metagenomic analysis performed in this study resulted in the identification of a *M. harundinacea* subsp., MAG_279, which appears to be a halotolerant acetoclastic methanogen with the flexibility to function at low (5 g/L Na^+^) and high salinity conditions (20 g/L Na^+^). Interestingly, its relative abundance was more than doubled when shifting from 5 to 20 g/L Na^+^, outcompeting the other dominant methanogen, *Methanothrix*_A MAG_280 for over 200 days of anaerobic reactor operation. Under the conditions described for the UASB reactors in this study, *M. harundinacea* MAG_279 appeared to drive saline granulation by secretion of complex EPS structures, of which the glycoconjugate patterns varied between low and high salinity probably due to the presence of multiple copies of the OST AglB. EPS secretion by *M. harundinacea* has never been reported before, to the best of our knowledge, while the presence of an acyl homoserine lactone (AHL)-based quorum sensing mechanism for this species (14) indicates that it has a role in triggering self-aggregation, and thus granulation. The genomic evidence presented in this study adds support to our previous observations that *Methanothrix* spp. can produce the osmolyte N^ε^-acetyl-β-lysine (41), which is again a novel reported attribute for this genus. N^ε^-acetyl-β-lysine is a rare osmolyte, the synthesis of which can be induced by a salinity increase similar to such conditions that trigger shifts in EPS glycoconjugate composition. Thus, future research is needed on the protein expression patterns activated under the above-mentioned environmental conditions for EPS secretion, glycoconjugate pattern shifts and the production of osmolytes within anaerobic bioreactors. The metabolic potential to synthesize the commercially valuable isoprenoid-precursors as IPP and isoprenoids as GGPP by both MAG_279 and MAG_280 could be seen as an added value when upscaling UASB processes with *Methanothrix*-enriched granules, in addition to their utility in coping with salinity stress. Indeed, in addition to renewable methane generation, anaerobic processes could be potentially utilized as biofactories, where value-added compounds can be produced at significant concentrations and then recovered from excess waste biomass (94). This proposition warrants a more detailed investigation of the economic feasibility and efficiency under different reactor operating configurations. Finally, this improved understanding of the mechanisms in which *Methanothrix* spp. are able to adapt to perform high-efficiency methanisation of organic waste at high salinities opens the door for further valorization of many wastewater streams that have previously been considered problematic.

## MATERIALS AND METHODS

### Bioreactor operation and sample collection.

Two lab-scale UASB reactors were operated for 217 days at a temperature of 35 ± 1°C to treat synthetic wastewater with different salinities: 5 g Na^+^/L, in the LS reactor, and 20 g Na^+^/L in the HS reactor. The start-up inoculum was a salinity adapted biomass (~8 g/L Na^+^) originating from the full-scale UASB reactor of the Shell plant in Moerdijk, the Netherlands, treating acetic/benzoic acid-rich wastewater. Details on the reactor operation are reported in Sudmalis, Gagliano, et al. (29) and in Table S1. Fresh granules were sampled at 10 time points from the sludge bed of the UASBs after granules had formed (Fig. S1), and stored at −20°C until DNA extraction.

### DNA extraction and metagenomic sequencing.

Genomic DNA was extracted from ~500 mg of granules using the FastDNA SPIN kit for soil (MPBio, USA) according to the manufacturer’s instructions. Prior the extraction, granules were washed with phosphate-buffered saline (15 min, 37° C), and then pre-treated by sonication (40 kHz, 50 W, 30 s) to disrupt the EPS matrix and facilitate the subsequent cell lysis. After extraction, the DNA concentration and purity were measured with the NanoDrop spectrophotometer (Thermo Fisher Scientific, Germany). Due to residual guanidine salts from the extraction kit, an additional purification procedure was performed. First, one volume of phenol:chloroform:isoamyl alcohol (25:24:1, Sigma-Aldrich, Germany) was mixed with the sample and centrifuged for 15 min at 13,000 × *g*. The recovered supernatant was then treated with RNase (RNase A, Promega, USA) following the manufacturer’s protocol. DNA precipitation was performed by adding sodium acetate 3M (1:10 vol/vol) plus two volumes of 70% cold ethanol, and kept at −20° C for 3 h. The recovered DNA pellet was washed with 70% ethanol twice, and then air-dried. Finally, samples where re-suspended in Tris-EDTA (pH 8), and the DNA concentration was measured using the Qubit dsDNA HS assay kit and the Qubit Fluorometer (Thermo Fisher Scientific, USA) following the manufacturer’s instructions. DNA samples were shipped to the DOE Joint Genome Institute (JGI), where they were sequenced using the Illumina NovaSeq platform in paired 151 bp read mode, with an average insert size of 279 bp. The average size of the raw metagenomes was 21 ± 3 Gbp (Table S5).

### Fluorescence microscopy.

Fluorescence *in situ* hybridization (FISH) was carried out on fixed granules collected from both reactors at the end of the reactor operation period by using ARC915 (domain *Archaea*) and MSMX860 (order *Methanosarcinales*) probes to visualize *Methanothrix* cells. Fresh granules collected from LS and HS reactors were used for the lectin staining analysis to visualize EPS structures, by applying 78 different lectins. FISH and lectin staining procedures, and the details of the epifluorescence and confocal laser scanning microscopy (CLSM) analyses are fully described in Gagliano et al. (32).

### Read quality filtering and de novo metagenome assembly.

Raw metagenome reads were quality filtered using BBDuk (v 38.34) (http://bbtools.jgi.doe.gov) to trim adapter sequences and right quality trim reads where the quality score dropped to 0. BBDuk was also used to remove reads that contained four or more “N” bases, had an average quality score less than 3 across the read, or had a minimum length of 33% of the full read length. Reads that mapped with BBMap to masked human, cat, dog, and mouse references at 93% identity, as well as to common microbial contaminants, were removed according to the JGI Metagenome Workflow (95). Quality-filtered interleaved reads for all time point samples from both the HS and LS reactor were separately co-assembled (by reactor) into contigs using MEGAHIT (v1.1.1), with a minimum contig length of 1,000 bp (96). Quality-filtered reads were then mapped to the co-assembled contigs using BowTie2 (v2.3.4.3) (97) with default parameters, and the mapping output was converted to BAM files with SAMtools (v1.9) (98). Anvi’o (v5.5) (99) was used to generate a contigs database, which stores tetranucleotide frequencies for each contig and uses Prodigal (v2.6.3) (100) to identify open reading frames (ORFs). Subsequently, profile databases were created using Anvi’o (v5.5) for each sample, which hosts contig coverage information by parsing the mapping (BAM) files. The sample profile databases were then merged, and the contigs were clustered in Anvi’o using CONCOCT (v1.1.0) (101) to form genome bins. CheckM (v1.0.13) (102) was used to estimate completeness and redundancy of the genomes using single copy genes (SCGs). Bins with more than 75% completion were manually refined using the “*anvi-refine*” option in Anvi’o (v5.5) to generate MAGs. To obtain a dereplicated set of MAGs across both of the reactor sample sets, the refined MAGs from both co-assemblies were dereplicated using dRep (v2.3.2) (103) with a Mash (v2.2) (104) cluster threshold of 0.8 for primary clustering, and the remaining parameters as default values. Taxonomic classification of the dereplicated set of MAGs was performed using the “*classify*” workflow of GTDB-Tk (v0.3.1) (42), using the GTDB taxonomy (release 89) (48). The output results for the archaeal MAGs are reported in Supplementary Data File 2. Quality-filtered reads were then re-mapped to the dereplicated set of MAGs using BowTie2 with default parameters, and the mapping output was converted to BAM files using SAMtools to generate coverage profiles of the dereplicated MAGs across samples for both reactors. The coverage of each dereplicated MAG, and the total cumulative MAG coverage within a metagenome sample, were used to estimate the relative abundance of each MAG as “*coverage of MAG/total cumulative coverage of the sample*.” The dereplicated set of MAGs were imported into Anvi’o for phylogenomic tree generation using the programs “*anvi-get-sequences-for-hmm-hits*” and “*anvi-gen-phylogenomic-tree*.” These programs used MUSCLE (v3.8.31) (105) to align homologous genes and FastTree (v2.1.10) (106) to generate a Newick tree. Plots for MAG completion and redundancy were generated using the tidyverse package in R (v3.6.3). Phylogenomic trees were plotted using ggtree package (v2.0.2) (107) and heatmaps were in generated using tidyverse package in R.

### Gene annotation and pathway prediction.

The MicroScope annotation platform (108) was used to annotate the ORFs in the recovered MAGs, and the annotations were then used as the input to reconstruct metabolic pathways using Pathway Tools (v23.5) (109) and the BioCyc and MetaCyc databases (110, 111). In Pathway Tools, the pathway prediction algorithm PathoLogic was applied to infer pathways in the annotated MAGs. Further identification of genes that were homologous to closely-related genomes, as well as genomes of organisms with potential salinity tolerance, was performed using BLASTP. Briefly, the amino acid sequences of genes encoding for N^ε^-acetyl- β-lysine and ectoine synthesis and the N-glycosylation pathway were selected from Methanosarcina mazeii Go1, Methanococcus maripaludis C5, and Haloferax volcanii Ds2 representative genomes (from NCBI database). For the mannose synthesis pathway, amino acid sequences for annotated mannose-6P-isomerase genes in *Methanothrix* spp., along with the characterized gene from Pyrococcus horikoshii OT3, were obtained from NCBI (Table S6). These reference sequences were used to make individual databases for searches using blast+ (v. 2.11.0), and then the “BLASTP” program was used to query homologous genes within the genomes of interest using an e-value threshold of 10^−4^. The positive BLASTP hits for mannose-6P-isomerase genes were further annotated using KofamKOALA (112) and the Pfam protein database (113). Homologous genes encoding for isoprenoid synthesis were searched using hidden Markov models with reference sequences from *Haloferax* members that encoded for phytoene synthase and phytoene desaturase. Briefly, the selected sequences were aligned in a clustal format using MUSCLE (v. 3.8.1551) (105) and a profile HMM was created using HMMER (v3.1b2) (114). The sequences were then searched using this profile HMM via the program “hmmsearch.” A threshold value of 10^−4^ was used to infer homology to search amino acid-translations of ORFs within the MAGs of interest. Inferred pathways were then manually curated in Pathway Tools to verify predictions from PathoLogic.

### Pangenome analysis.

A pangenome analysis (Fig. 1) was conducted by comparing the three *Methanothrix*-related MAGs with the NCBI genomes of two representative *Methanothrix harundinacea*, Methanosarcina mazei Go1, Methanococcus maripaludis C5, and Haloferax volcanii Ds2, via the anvi’o pangenomics workflow outlined in Delmont and Eren (115). Briefly, an anvi’o genome database (“*anvi-gen-genomes-storage*”) was generated to store DNA and amino acid sequences, and functional annotations of the genomes surveyed. The pangenome was computed (using “*anvi-display-pan*”) to identify gene clusters based on amino acid sequence similarity, and the anvi’o interface was used to search and highlight genes of interest based on the predicted function obtained via genome annotation.

### Data availability.

NCBI BioProject ID and JGI IMG IDs for the metagenomes are provided in Table S7. All of the archaeal MAGs reported in Fig. 2 were deposited in the NCBI database, and the accession numbers are reported in Table S8.
