# Supplementary material for: Functional Insights of Salinity Stress-Related Pathways in Metagenome-Resolved Methanothrix Genomes
Source: Appl Environ Microbiol. 2022 Apr 28;88(10):e02449-21. doi: 10.1128/aem.02449-21 (PMC9128505; doi:10.1128/aem.02449-21)
Supplement: Supplemental file 3 — Fig. s1 to S9 and Tables S1 to S8. Download aem.02449-21-s0001.pdf, PDF file, 1.9 MB [file aem.02449-21-s0001.pdf]

SUPPLEMENTARY INFORMATION

Functional insights of salinity stress-related pathways in metagenome-resolved *Methanothrix* genomes

Maria Cristina Gagliano [1,2], Pranav Sampara [3], Caroline M. Plugge [1,2], Hardy Temmink [1,4], Dainis Sudmalis [4] and Ryan Ziels [3]

(1) Wetsus – European Centre of Excellence for Sustainable Water Technology, Oostergoweg 9, 8911MA Leeuwarden, the Netherlands
(2) Laboratory of Microbiology, Wageningen University and Research, Stippeneng 4, 6708 WE, Wageningen, the Netherlands
(3) Civil Engineering, University of British Columbia, 2002 - 6250 Applied Science Lane, Vancouver, BC, V6T 1Z4, Canada
(4) Department of Environmental Technology, Wageningen University and Research, Bornse Weilanden 9, 6708 WG, Wageningen, the Netherlands

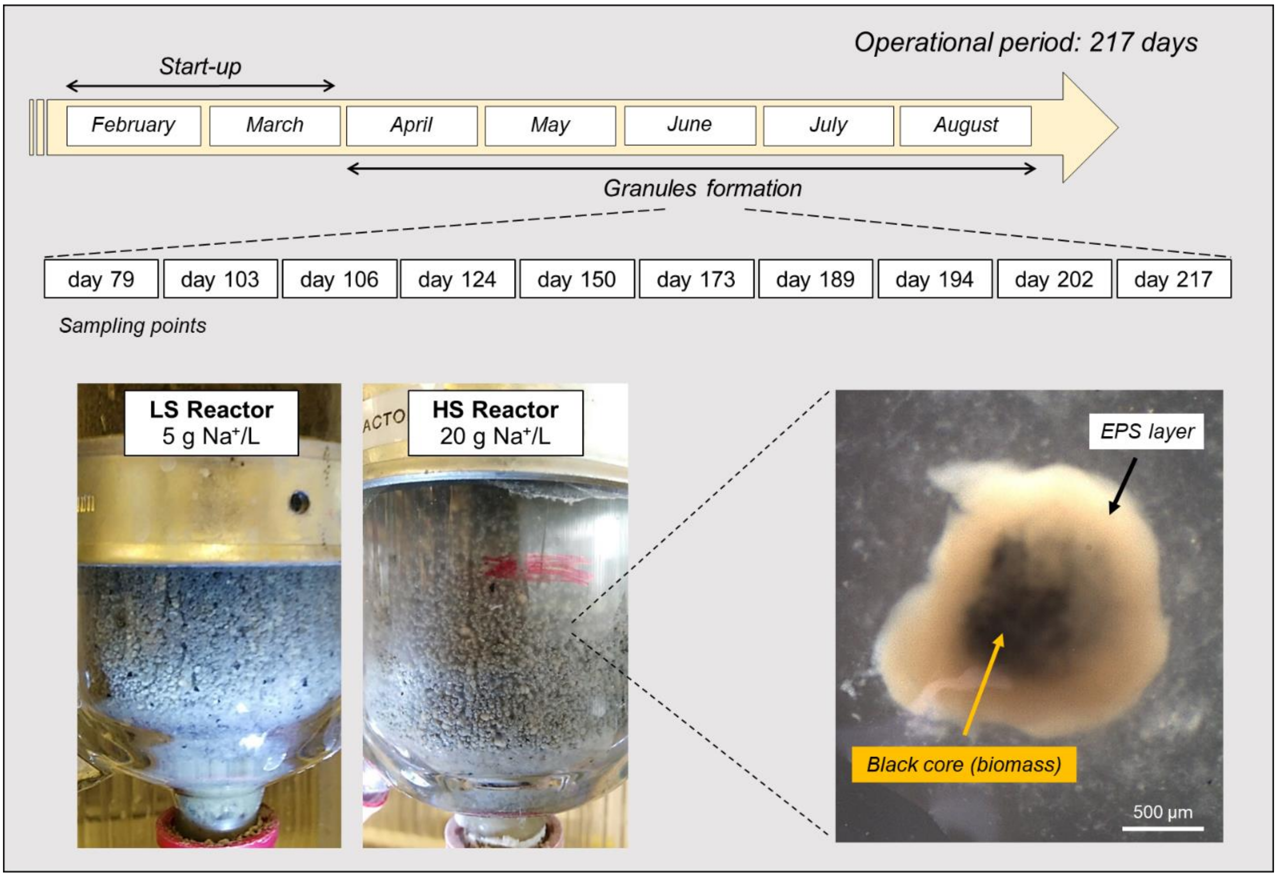

Figure S1 – The ten sampling points for the DNA extraction along the 217 days process of the two UASB reactors. Immediately after the increase of the OLR from 7 to 12 g COD/L-d (around day 79), granule development occurred in the HS reactor (20 g/L of Na^+). The microbial population was then monitored from day 79 until the end of the process (day 217), where big, EPS-rich granules were observed in both reactors (see above images). Further details on the structural development of granules over time and the related microbial community morphology and identity can be found in Gagliano *et al.*, 2018, 2020.

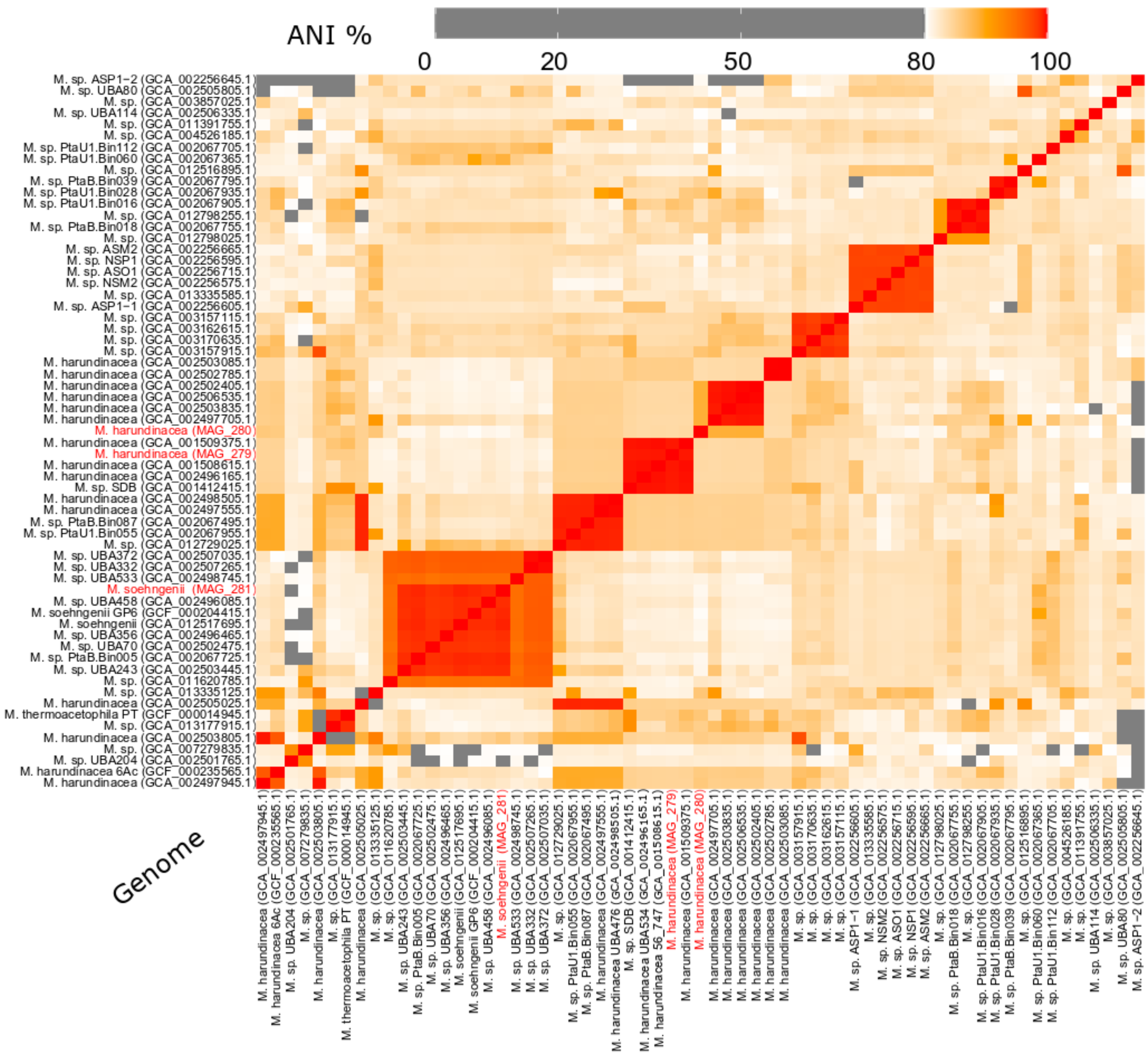

Figure S2 - Average nucleotide identity heatmap of 60 *Methanothrix* genomes (NCBI accession numbers in parentheses) and the three *Methanothrix* MAGs from this study (marked in red). The heatmap was generated using ANIm method in pyANI (v.0.2.10) (3).

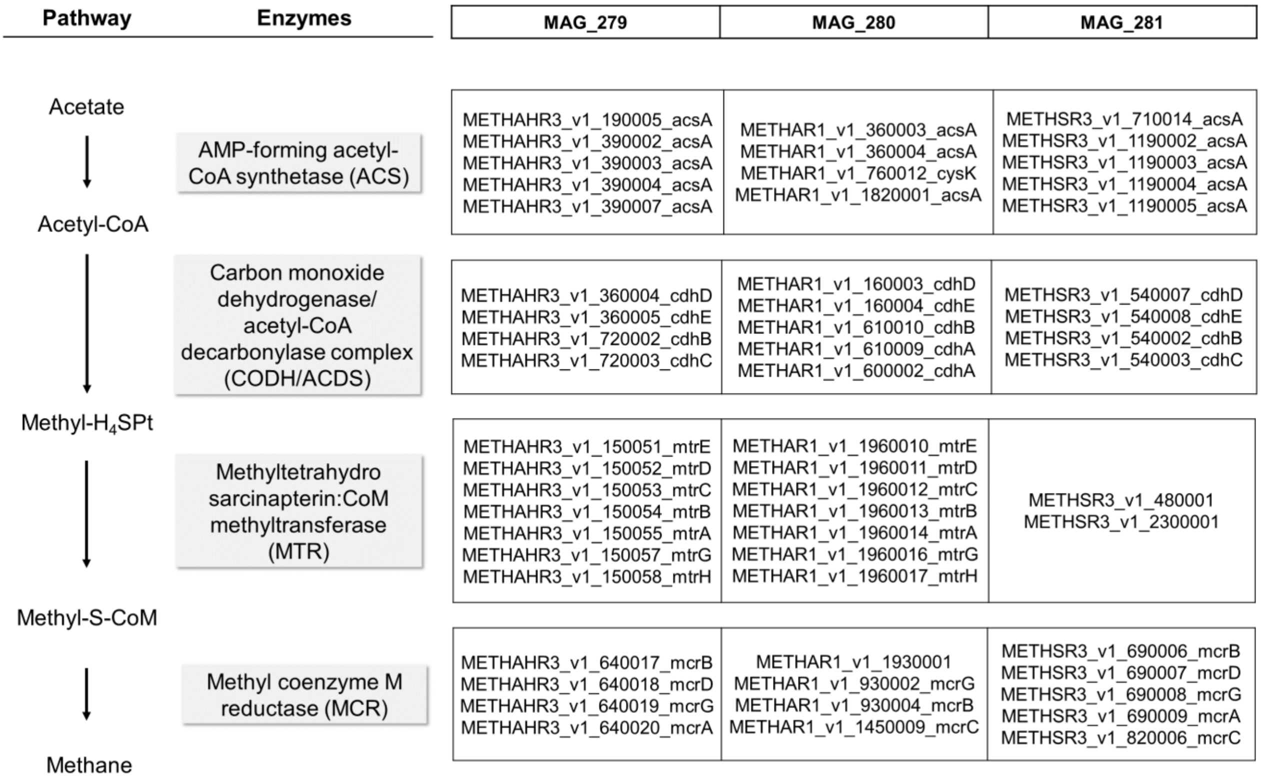

Figure S3 - Pathway for acetoclastic methanogenesis as identified in *M. harundinacea* MAG_279, *Methanothrix_A* MAG_280 and *M. soehgenii* MAG_281. In the grey boxes, the enzymes catalysing the pathway, in the table on the right, the encoding genes identified per each MAGs. All enzymes catalysing the pathway are present in the three *Methanothrix* MAGs.

As observed in *Methanothrix thermophila*, the three MAGs have several copies of the *acs* gene, of which some are tandemly positioned. This indicates that differential *acs* expression could provide *Methanothrix* with the ability to adapt to changes in its environment (4).

According to the same authors, the presence of four ACSs in addition to multiple putative acetylases and deacetylases in *M. thermophila* suggests that acetate activation and its regulation are much more complex than expected.

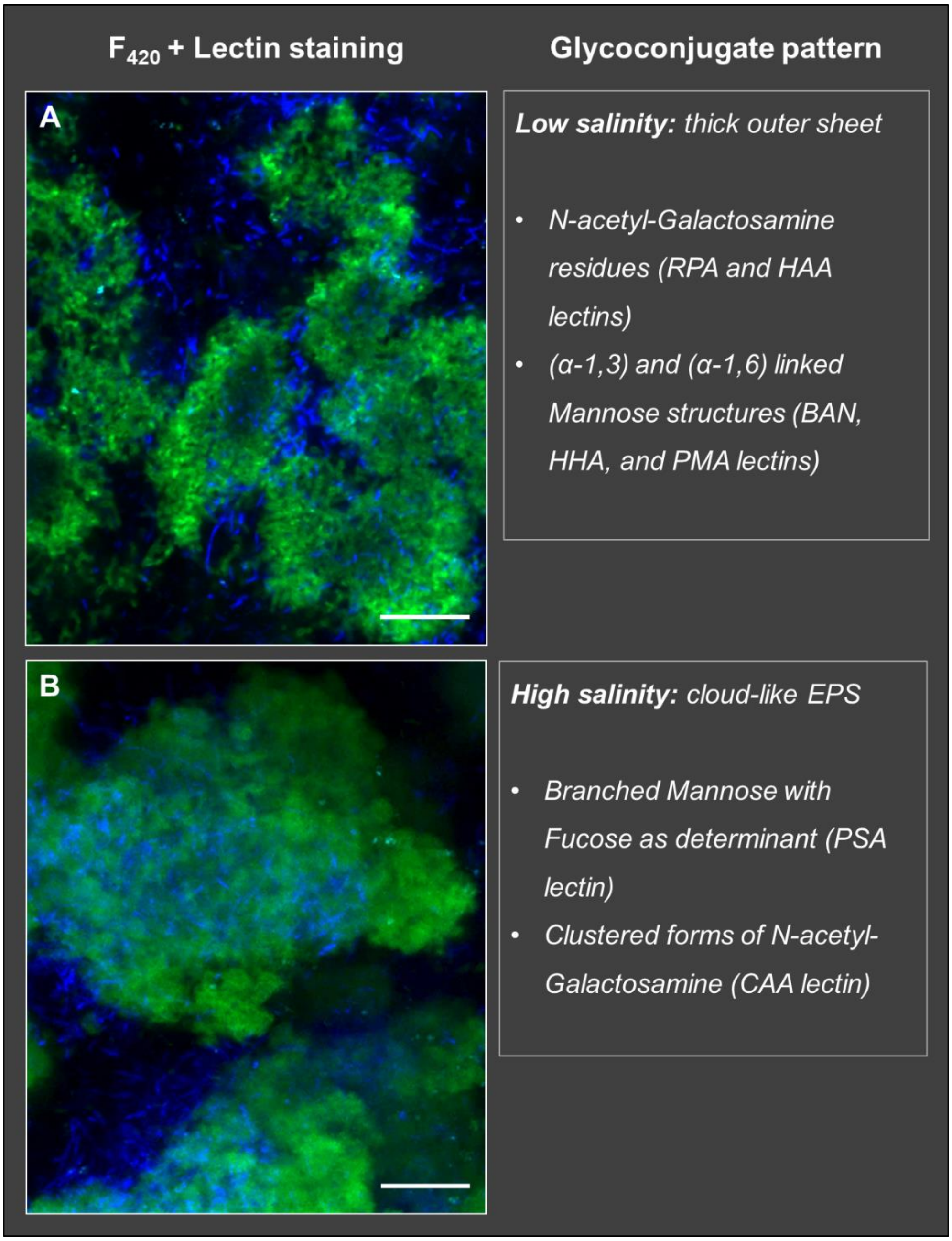

Figure S4 – CLSM maximum average projections of the two main EPS structures surrounding the round-shaped methanogenic clusters (identified via their F_{420} autofluorescence, in blue), detected at low (A) and high salinity (B) conditions. The relative glycoconjugate patterns per each EPS structure is reported on the right, and it was revealed after Fluorescence Lectin Bar Coding (FLBC) analysis with 78 different lectins targeting different sugars/conformations. Size bar is 20 µm. More information about the FLBC analysis carried out on saline granules is reported in Gagliano et al. (2018).

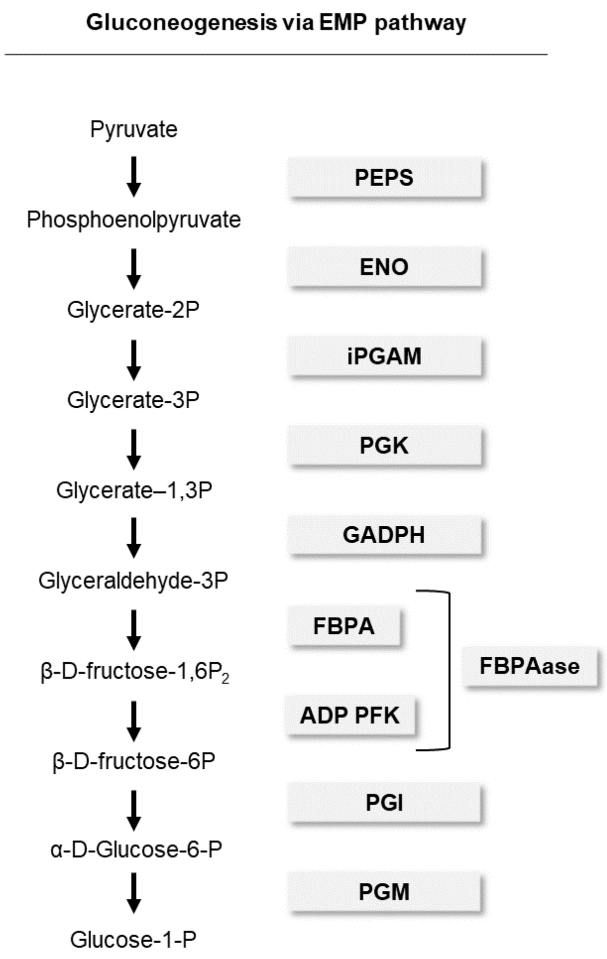

	Genetic loci			
**Gluconeogenesis enzymes**	***Methanococcus maripaludis* DSM 2067 (NZ_CP026606.1)**	***M. harundinacea* 6Ac (NC_017527.1)**	***M. harundinacea* MAG_279**	***Methanothrix_A* MAG_280**
PEPS: PEP synthetase	MMJJ_RS08695 (WP_104838478.1)	MHAR_RS03915 (WP_014586315.1)	METHAHR3_v1_20<br>042	METHAR1_v1_182<br>0007
ENO: Enolase (or phosphopyruvate hydratase)	MMJJ_RS03190 (WP_104837655.1)	MHAR_RS10600 (WP_014587611.1)	METHAHR3_v1_68<br>0001	METHAR1_v1_164<br>0006
iPGAM: 2,3 bisphosphoglycerate cofactor independent phosphoglycerate mutase	MMJJ_RS04825 (WP_104838587.1)<br>MMJJ_RS06955 (WP_104838224.1)	MHAR_RS02730 (WP_014586095.1)	METHAHR3_v1_65<br>0005	METHAR1_v1_145<br>0011
dPGAM: 2,3-bisphosphoglycerate cofactor dependent mutase		MHAR_RS00330 (WP_048144181.1)	METHAHR3_v1_60<br>003	METHAR1_v1_800<br>13
PGK: phosphoglycerate kinase	MMJJ_RS06485 (WP_013999848.1)	MHAR_RS06795 (WP_014586871.1)	METHAHR3_v1_60<br>0034	METHAR1_v1_134<br>0002
GADPH: glyceraldehyde-3-phosphate dehydrogenase	MMJJ_RS03640 (WP_104837728.1)	MHAR_RS06365 (WP_143763321.1)	METHAHR3_v1_20<br>018	METHAR1_v1_700<br>022
FBPA: fructose-1,6-bisphosphate aldolase	MMJJ_RS01460 (WP_104837366.1)<br>MMJJ_RS03800 (WP_104837752.1)	MHAR_RS11505 (WP_014587791.1)	METHAHR3_v1_51<br>0050	METHAR1_v1_147<br>0012
FBPAase: fructose-bisphosphatase	MMJJ_RS03680 (WP_013998745.1)	MHAR_RS08565 (WP_014587218.1)	METHAHR3_v1_25<br>0003	METHAR1_v1_129<br>0002
ADP PFK/GLK: ADP-dependent phosphofructokinase/glucokinase	MMJJ_RS07675 (WP_104838310.1)			
PGI: glucose-6-phosphate isomerase	MMJJ_RS07680 (WP_104838311.1)	MHAR_RS07680 (WP_048144925.1)	METHAHR3_v1_10<br>063	METHAR1_v1_163<br>0007
PGM: phosphoglucomutase	MMJJ_RS07280 (WP_104838257.1)	MHAR_RS10515 (WP_081472332.1)	METHAHR3_v1_33<br>0040	METHAR1_v1_173<br>0005

Figure S5 - The enzymes of gluconeogenesis via the Embden-Meyerhof-Parnas (EMP) pathway (depicted on the left) identified in *M. maripaludis* DSM 2067 crude extracts (5) where used to identify the corresponding genetic loci in *M. harundinacea* 6Ac, and then in MAG_729 and MAG_280 via BLASTP. The dPGAM function, interchangeable with iPGAM, is found mostly in bacteria and eukaryotes, and only few archaea (as *Methanosarcina* ssp.). Differently than *M. maripaludis*, the three *Methanothrix* sp. do not possess an ADP-dependent phosphofructokinase function, which is not fundamental for gluconeogenesis as far as the FBPAase enzyme is present.

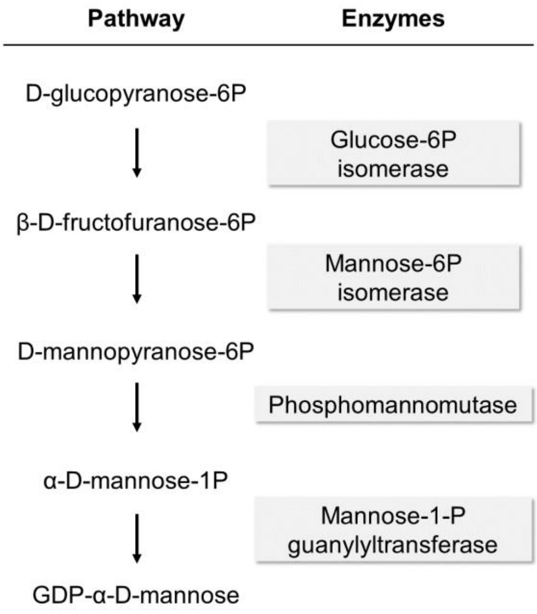

MAG_279	MAG_280
METHAHR3_v1_10063	METHAR1_v1_1630007
METHAHR3_v1_520007	METHAR1_v1_1180007
METHAHR3_v1_33040<br>METHAHR3_v1_600028	METHAR1_v1_1730005<br>METHAR1_v1_1340007
METHAHR3_v1_600028	METHAR1_v1_1340007

Figure S6 - Pathway for mannose synthesis as identified in *Methanothrix harundinacea* MAG_279 and *Methanothrix_A* MAG_280 with the corresponding encoding genes identified per each MAGs.

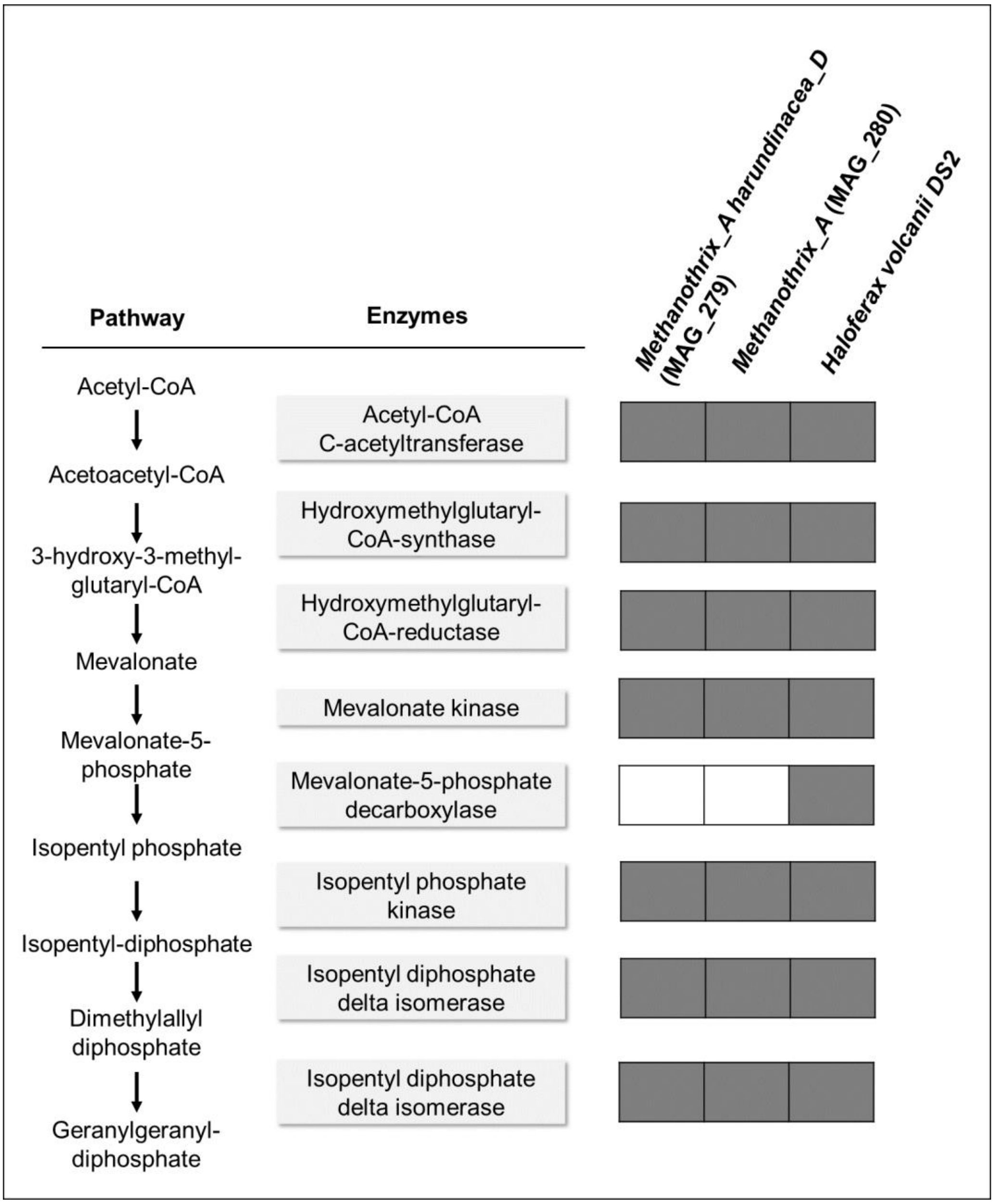

Figure S7 - Synthesis of geranylgeranyl diphosphate (GGPP) via the mevalonate pathway as observed in the genome of *Haloferax volcanii* DS2 (accession number: CP001956.1), in comparison to the salinity adapted MAGs (MAG_279 and MAG_280). *M. harundinacea* MAG_279 and *Methanothrix_A* MAG_280 lack the mevalonate-5-phosphate decarboxylase enzyme, thus they have alternative enzymes to cover the function, as observed in the archaeon *Aeropyrum pernix* (see Figure 5).

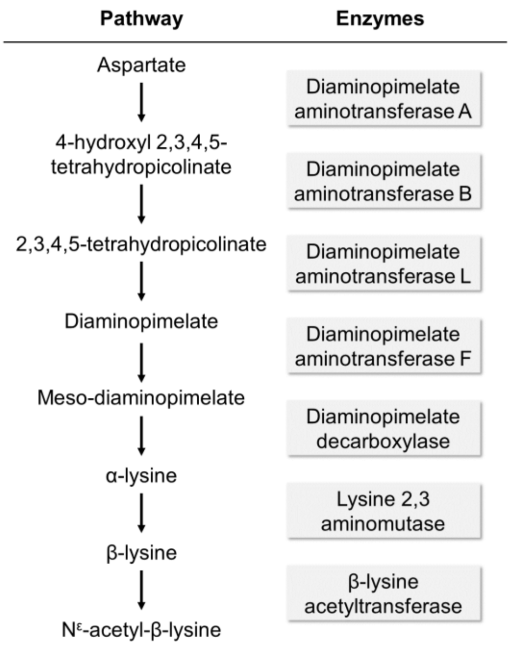

MAG_279	MAG_280	MAG_281
METHAHR3_v1_380004	METHAR1_v1_1240018	METHSR3_v1_1340010
METHAHR3_v1_380003	METHAR1_v1_1240017	METHSR3_v1_1340009
METHAHR3_v1_330035	METHAR1_v1_1850003	METHSR3_v1_2320004
METHAHR3_v1_620005	METHAR1_v1_1590013	METHSR3_v1_990016
METHAHR3_v1_230033_	METHAR1_v1_690002	METHSR3_v1_2380009
METHAHR3_v1_50049<br>METHAHR3_v1_710006	METHAR1_v1_680001<br>METHAR1_v1_1500004	METHSR3_v1_1420003
METHAHR3_v1_30015	METHAR1_v1_480023	

Figure S8 - Pathway for N^{ε}-acetyl- β-lysine synthesis as identified in *Methanothrix harundinacea* MAG_279, *Methanothrix_A* MAG_280 and *M. soehngenii* MAG_281. In the grey boxes, the enzymes catalysing the pathway, and in the table on the right the annotated genes per each function, identified by homology to genes of *Methanosarcina mazeii* Go1 (accession number AE008384) and *Methanococcus maripaludis* C5 (accession number CP000609) via a BLASTP search with an e-value threshold of 10^{-4}. A blank cell in the table indicates the lack of the gene encoding for the enzyme.

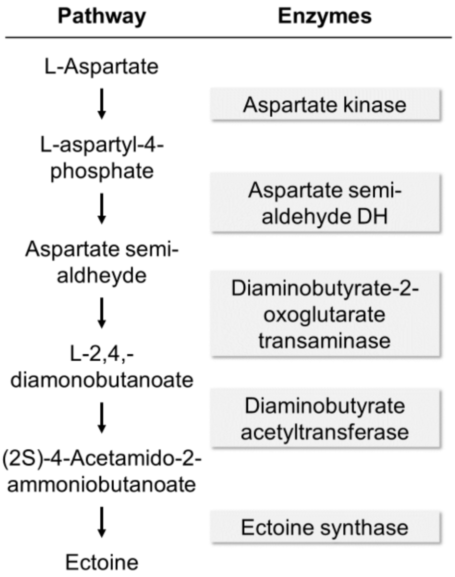

MAG_279	MAG_280	MAG_281
METHAHR3_v1_770003	METHAR1_v1_700027	METHSR3_v1_1610004
METHAHR3_v1_280038_	METHAR1_v1_260006	METHSR3_v1_1580015
METHAHR3_v1_300004	METHAR1_v1_670001	METHSR3_v1_1960002
METHAHR3_v1_300005		METHSR3_v1_1960001
METHAHR3_v1_300003	METHAR1_v1_670002	METHSR3_v1_1960003

Figure S9 - Pathway for ectoine synthesis as identified in *Methanothrix harundinacea* MAG_279, *Methanothrix_A* MAG_280 and *M. soehngenii* MAG_281. In the grey boxes, the enzymes catalysing the pathway, and in the table on the right the annotated genes per each function, by homology to genes *Methanosarcina mazeii* Go1 (accession number AE008384) and *Methanococcus maripaludis* C5 (accession number CP000609) via a BLASTP search with an e-value threshold of 10^{-4}. A blank cell in the table indicates the lack of the gene encoding for the enzyme.

Table S1 – UASB Reactors operation and main performances during the 217 days of process. More details are reported in Sudmalis et al., (2018).

	**LS Reactor**	**HS Reactor**
Operating Temperature	35 ± 1 ˚C	35 ± 1 ˚C
Start-up Inoculum	6 g VSS/L	6 g VSS/L
Salinity	5g Na^+/L	20g Na^+/L
Loading rate during start - up	1g COD/L·d	1g COD/L·d
*Increase of COD loading rate on day 31**	3.7 g COD/L·d	3.8 g COD/L·d
*Increase of influent COD loading rate to final values on day 52**	11 g COD/L·d	12 g COD/L·d
Upflow velocity at final loading rate	1 m/h	1 m/h
Average Biogas Methane content	63.6 ± 4.6%	68.4 ± 4.3%
Average COD removal efficiencies	97.2 ± 2.1%	94.0 ± 2.2%

**Increase of influent loading rate corresponds to increasing influent COD from 3 to 7 and from 7 to 12 g/L*

Table S2 – Putative N-glycosylation genes detected in *Methanothrix harundinacea* MAG_279 and *Methanothrix_A* MAG_280. The genes are identified from MAG annotation via MicroScope annotation platform and by homology to genes in the N-glycosylation cluster in *Haloferax volcanii* Ds2 (NCBI accession number: GCF_000025685.1) and *Methanococcus maripaludis* C5 (NCBI accession number: GCF_000016125.1) via a BLASTP search with an e-value threshold of 10^{-4}.

	Oligosccharyl transferase	***Methyl transferase***	***Glycosyl transferase***	***Flippase***	***Epimerase***
**MAG_279**	METHAHR3_v1_200012	METHAHR3_v1_330039	METHAHR3_v1_330041	METHAHR3_v1_490049	METHAHR3_v1_630008
	METHAHR3_v1_580020	METHAHR3_v1_200013	METHAHR3_v1_120038		METHAHR3_v1_800014
	METHAHR3_v1_630003	METHAHR3_v1_710024	METHAHR3_v1_120039		
	METHAHR3_v1_750006		METHAHR3_v1_30018		
			METHAHR3_v1_30021		
			METHAHR3_v1_490047		
			METHAHR3_v1_490050		
			METHAHR3_v1_630004		
			METHAHR3_v1_630005		
**MAG_280**	METHAR1_v1_220001	METHAR1_v1_1730004	METHAR1_v1_1730006	METHAR1_v1_1750006	METHAR1_v1_1130002
	METHAR1_v1_470002	METHAR1_v1_820009	METHAR1_v1_1610008	METHAR1_v1_820003	
	METHAR1_v1_660015		METHAR1_v1_1610011		
			METHAR1_v1_1750004		
			METHAR1_v1_1750005		
			METHAR1_v1_1750008		
			METHAR1_v1_1800009		
			METHAR1_v1_820001		
			METHAR1_v1_820002		

Table S3 - Identity of nucleotide and protein sequences of each AglB gene detected in *M. harundinacea* MAG_279 and *Methanothrix* MAG_280.

	BLAST N			Blast P - DELTA BLAST			
***M. harundinacea* MAG_279**	**Aligned sequence**	**Identity**	**Query cover**	**Aligned sequence**	**Identity**	**Query cover**	**Protein features**
METHAHR3_v1_200012	OST, STT3 subunit [*M. harundinacea* 6Ac] (AET63921.1)	77.5%	99%	OST, STT3 subunit [*M. harundinacea*] (KUK43611.1)	99.7%	100%	STT3 and PMT_2 subunits
METHAHR3_v1_580020	OST, STT3 subunit [*M. harundinacea* 6Ac] (AET64803.1)	75%	60%	OST, STT3 subunit [*M. harundinacea*] (KUK44794.1)	99.6%	100%	STT3 and PMT_2 subunits
METHAHR3_v1_630003 (*)	OST, STT3 subunit (**) [*M. harundinacea* 6Ac] (AET64459.1)	73.4%	100%	OST, STT3 subunit [*M. harundinacea*] (KUK94790.1)	90%	100%	STT3 subunit
METHAHR3_v1_750006	OST, STT3 subunit [*M. harundinace* a 6Ac] (AET64459.1)	71.3%	91%	hypothetical protein APR56_06270(**) [*Methanothrix* sp. SDB] (KQC12563.1)	100%	100%	STT3 and PMT_2 subunits

	BLAST N			Blast P- DELTA BLAST			
***Methanothrix* MAG_280**	**Aligned sequence**	**Identity**	**Query cover**	**Aligned sequence**	**Identity**	**Query cover**	**Protein features**
METHAR1_v1_660015	OST, STT3 subunit [*M. harundinacea* 6Ac] (AET65088.1)	69.3%	99%	OST, archaeosortase A system-associated [*Methanosarcina siciliae*] (WP_048173653.1)	24.5%	100%	STT3 and PMT_2 subunits
METHAR1_v1_470002	OST, STT3 subunit [*M. harundinacea* 6Ac] (AET63921.1)	76.7%	88%	hypothetical protein APR56_06270(**) [*Methanothrix* sp. SDB] (KQC12563.1)	39%	65%	STT3 and PMT_2 subunits
METHAR1_v1_220001	OST, STT3 subunit [*M. harundinacea* 6Ac] (AET63921.1)	77.2%	100%	Glycosyltransferase AglB [*Methanothrix* sp. PtaU1.Bin055] (OPY56061.1)	73%	98%	STT3 subunit

(*) this gene is probably partially reconstructed, with a gap in between
(**) this is wrongly annotated just as hypothetical protein, because it contains both STT3 and PMT2 domains

Table S4 – Putative orthologs of the enzymes of Aeropyrum-type, modified MVA pathway as identified by Hakayawa et al., (2018) for *M. thermophila*, and in this study by analyzing the genomes of *M. harundinacea* 6Ac, *M. harundinacea* MAG_279 and *Methanothrix_A* MAG_280 via NCBI BLAST N.

	**Aeropyrum pernix enzimes**			
	**AcnX-1**	**AcnX-2**	**UbiD-like**	**UbiX-like**
***M. thermophila* PT**	MTHE_0207	MTHE_0208	MTHE_0940	MTHE_0501
annotated function	DUF521 domain-containing protein	DUF126 domain-containing protein	UbiD family decarboxylase	UbiX family flavin prenyltransferase
***M. harundinacea* 6Ac**	MHAR_1472	MHAR_1471	MHAR_2380	MHAR_1408
annotated function	Putative aconitase	Hypothetical protein	UbiD family decarboxylase	Phenylacrylic acid decarboxylase
***M. harundinacea* MAG_279**	METHAHR3_v1_620018	METHAHR3_v1_620017	METHAHR3_v1_290015	METHAHR3_v1_480011
annotated function	Putative aconitase subunit I	Aconitate hydratase X	3-polyprenyl-4-hydroxybenzoate-carboxylase	3-octaprenyl-4-hydroxybenzoate-carboxylase
***Methanothix_* A MAG_280**	METHAR1_v1_520040	METHAR1_v1_520039	METHAR1_v1_560032	METHAR1_v1_1060035
annotated function	Putative aconitase subunit I	Aconitate hydratase X	3-polyprenyl-4-hydroxybenzoate-carboxylase	Flavin prenyltransferase UbiX

Table S5 - Number of bases in the raw metagenomes. The average size of the raw metagenomes was 21275250564 ± 3103732620 bp.

Reactor	Sample name	Sampling day	Metagenome size
Low salinity (LS)	15_04	79	22266631536
	09_05	103	25417402670
	12_05	106	26312647242
	31_05	124	21879854398
	27_06	150	22152314268
	18_07	173	22623254880
	3_08	189	21838279568
	8_08	194	21601356038
	15_08	202	14395120222
	31_08	217	24133079720
High salinity (HS)	15_04	79	21307201696
	09_05	103	21767905112
	12_05	106	17644836522
	31_05	124	21533391844
	27_06	150	15740307346
	18_07	173	19931607098
	3_08	189	25009386174
	8_08	194	17772149152
	15_08	202	19037350368
	31_08	217	23140935428

Table S6 - The amino acid protein sequences used to construct the BLASTP database for mannose-6P-isomerase genes for querying the reconstructed MAGs from this study.

Genome	NCBI protein accession	Annotation on NCBI
Pyrococcus horikoshii	WP_010885015.1	mannose-1-phosphate guanylyltransferase/mannose-6-phosphate isomerase
Methanothrix sp. PtaB.Bin039	OPX77532.1	Mannose-6-phosphate isomerase
Methanothrix harundinacea	WP_048144846.1	cupin domain-containing protein
Methanothrix harundinacea	WP_014586887.1	cupin domain-containing protein
Methanothrix sp. PtaU1.Bin112	OPY54891.1	Cupin domain protein
Methanothrix sp. PtaU1.Bin112	OPY53436.1	Cupin domain protein
Methanothrix sp. PtaU1.Bin055	OPY53403.1	Cupin domain protein
Methanothrix sp. PtaU1.Bin060	OPY49953.1	Cupin domain protein
Methanothrix sp. PtaU1.Bin016	OPY46112.1	Cupin domain protein
Methanothrix sp. PtaU1.Bin028	OPY44753.1	Cupin domain protein
Methanothrix sp. PtaB.Bin087	OPX80409.1	Cupin domain protein
Methanothrix sp. PtaB.Bin039	OPX80043.1	Cupin domain protein
Methanothrix sp. PtaB.Bin087	OPX79092.1	Cupin domain protein
Methanothrix sp. PtaB.Bin018	OPX75711.1	Cupin domain protein
Methanosaeta harundinacea	KUK97713.1	Uncharacterized protein XE07_0127
Methanosaeta harundinacea	KUK45367.1	Uncharacterized protein XD72_0270
Methanosaeta harundinacea 6Ac	AET64702.1	hypothetical protein Mhar_1338
Methanosaeta harundinacea 6Ac	AET64330.1	Cupin domain protein

Table S7 - NCBI Project ID and JGI IMG ID for the samples from both reactors.

Reactor	Sample name	Sampling day	NCBI BioProject Accession	JGI IMG Project ID
Low salinity (LS)	15_04	79	567718	3300033174
	09_05	103	567719	3300033177
	12_05	106	567720	3300033170
	31_05	124	567721	3300033178
	27_06	150	567722	3300033176
	18_07	173	567723	3300033169
	3_08	189	567724	3300033172
	8_08	194	567725	3300033173
	15_08	202	567727	3300034686
	31_08	217	567726	3300033175
High salinity (HS)	15_04	79	567728	3300033165
	09_05	103	567729	3300033161
	12_05	106	567730	3300033164
	31_05	124	567731	3300033171
	27_06	150	567732	3300033428
	18_07	173	655052	3300037528
	3_08	189	567733	3300033167
	8_08	194	567734	3300033163
	15_08	202	567662	3300033162
	31_08	217	567735	3300033166

Table S8 – List of the recovered archaeal MAGs above 75 % completion.

MAGs	Completeness (%)	Contamination (%)	GC content (%)	Genome length (bp)	Number of contigs	N50 (bp)	BioSample Accession ID	Genome accession number
**MAG_279**	88.49	0.66	57.2	1960038	81	36807	SAMN18737511	CP086252
**MAG_280**	93.42	0.66	59.2	2001575	187	14898	SAMN18737512	CP086218
**MAG_281**	93.46	2.29	52	2359115	246	12226	SAMN18737513	CP086219
**MAG_282**	76.38	2.8	43.8	2075090	328	7140	SAMN19322122	JAHFZE000000000
**MAG_283**	97.39	0.98	62	2558312	96	36186	SAMN19322288	JAHKLM000000000
**MAG_284**	80	0	60.5	2010371	123	22033	SAMN19322289	JAHKLN000000000
**MAG_285**	100	6.78	53.2	1924779	127	28164	SAMN19322290	JAHKLO000000000
**MAG_286**	89.39	0.8	37.9	1679755	142	19715	SAMN19322291	JAHKLP000000000
**MAG_287**	94.93	1.2	37.9	2213772	230	12401	SAMN19322292	JAHKLQ000000000
**MAG_288**	98.93	0	39.1	2470617	86	51890	SAMN19322293	JAHKLR000000000
**MAG_289**	84.49	2.4	33.7	1627106	233	7940	SAMN19322294	JAHKLS000000000
**MAG_290**	100	0	25.8	1663006	75	34665	SAMN19322295	JAHKLT000000000
**MAG_291**	95.2	0.8	25.4	2026875	74	42181	SAMN19322296	JAHKLU000000000
**MAG_293**	80.87	3.93	58.6	1513793	247	7080	SAMN19322297	JAHKLV000000000
**MAG_294**	93.87	4.21	35.5	1945541	102	36820	SAMN19322298	JAHKLW000000000
**MAG_296**	86.14	3.74	39.2	2122498	149	48117	SAMN19322299	JAHKLX000000000
**MAG_297**	81.93	0	28	828311	7	239484	SAMN19322300	JAHKLY000000000
**MAG_298**	83.18	0	34.3	1196794	60	73065	SAMN19322301	JAHKLZ000000000

References

1. Gagliano MC, Neu TR, Kuhlicke U, Sudmalis D, Temmink H, Plugge CM. 2018. EPS Glycoconjugate Profiles Shift as Adaptive Response in Anaerobic Microbial Granulation at High Salinity. Front Microbiol 9.

2. Gagliano MC, Sudmalis D, Pei R, Temmink H, Plugge CM. 2020. Microbial Community Drivers in Anaerobic Granulation at High Salinity. Front Microbiol 11.

3. Pritchard L, Glover RH, Humphris S, Elphinstone JG, Toth IK. 2015. Genomics and taxonomy in diagnostics for food security: soft-rotting enterobacterial plant pathogens. Anal Methods 8:12–24.

4. Smith KS, Ingram-Smith C. 2007. Methanosaeta, the forgotten methanogen? Trends Microbiol 15:150–155.

5. Yu JP, Ladapo J, Whitman WB. 1994. Pathway of glycogen metabolism in Methanococcus maripaludis. J Bacteriol 176:325–332.
